# New Insight to Structure-Function Relationship of GalNAc Mediated Primary Interaction between Insecticidal Cry1Ac Toxin and HaALP Receptor of *Helicoverpa armigera*

**DOI:** 10.1371/journal.pone.0078249

**Published:** 2013-10-24

**Authors:** Anindita Sengupta, Anindya Sarkar, Prerna Priya, Shubhra Ghosh Dastidar, Sampa Das

**Affiliations:** 1 Division of Plant Biology, Bose Institute, Kolkata, West Bengal, India; 2 Bioinformatics Centre, Bose Institute, Kolkata, West Bengal, India; University of Tennessee, United States of America

## Abstract

Over the last few decades Cry1Ac toxin has been widely used in controlling the insect attack due to its high specificity towards target insects. The pore-forming toxin undergoes a complex mechanism in the insect midgut involving sequential interaction with specific glycosylated receptors in which terminal GalNAc molecule plays a vital role. Recent studies on Cry toxins interactions with specific receptors revealed the importance of several amino acid residues in domain III of Cry1Ac, namely Q509, N510, R511, Y513 and W545, serve as potential binding sites that surround the putative GalNAc binding pocket and mediate the toxin-receptor interaction. In the present study, alanine substitution mutations were generated in the Cry1Ac domain III region and functional significance of those key residues was monitored by insect bioassay on *Helicoverpa armigera* larvae. In addition, ligand blot analysis and SPR binding assay was performed to monitor the binding characteristics of Cry1Ac wild type and mutant toxins towards HaALP receptor isolated from *Helicoverpa armigera*. Mutagenesis data revealed that, alanine substitutions in R511, Y513 and W545 substantially impacted the relative affinity towards HaALP receptor and toxicity toward target insect. Furthermore, *in silico* study of GalNAc-mediated interaction also confirmed the important roles of these residues. This structural analysis will provide a detail insight for evaluating and engineering new generation Cry toxins to address the problem of change in insect behavioral patterns.

## Introduction

The advancement of genetic engineering and continued success of transgenic plant development with eco-friendly insecticidal genes made remarkable contributions to the global food security. Transgenic plants expressing different insecticidal genes are good alternatives to harmful chemical insecticides [1] and also led to the worldwide acceptance of Bt crystal proteins, synthesized by *Bacillus thuringiensis*, as a major insecticidal toxin against different insects [2]. *B. thuringiensis*, a gram positive soil bacterium [3], produces a number of protoxin proteins during sporulation that are deposited in the form of parasporal crystals and known as Bt toxin or δ-endotoxin [4]. These environmental friendly insecticidal toxins are of great importance due to their potency and high specificity towards a wide range of insect pest groups, namely Lepidoptera, Coleopteran and Diptera [5]. The unique mode of action of Bt toxins towards target insects and lack of toxicity toward other organisms have greatly facilitated its widespread use in transgenic plant development and commercialization of various Bt transgenic plants.

Among them, Cry1Ac has made a significant contribution in controlling insects for major crops such as cotton, soybean, maize, chickpea etc [6]. A major breakthrough in this aspect was made possible by several groups including the present investigators, by developing transgenic plants with synthetic codon optimized *cry1Ac* gene [7,8]. The toxic effects of such transgenic plants were verified extensively on *Helicoverpa armigera*, a devastating pest that poses a severe worldwide economic threat for various crops [9]. It has been estimated that 25% of the crops are lost worldwide because of its voracious feeding behaviour, high reproductive rate, and polyphagous nature [10]. The selective nature of Cry1Ac toxin relies on the presence of specific receptors in the insect gut membrane. Binding of this toxin to these receptors is likely to be the most important criterion for the efficacy of an individual toxin molecule against a particular insect [11]. Thus, the specificity of the interaction between toxin and midgut receptors in insect brush border membrane vesicles (BBMV) determines the utility of the toxin molecule against the insect. Importance of these receptors become clearer from the fact that insects develop resistance often due to loss of specific binding capacity of the toxins either due to loss or modification of the receptors [12]. The crystal structure of Cry toxin has been resolved by X-ray crystallography that revealed a high structural similarity with three distinct domains [13].. The N-terminal Domain I consists of seven transmembrane α-helices, responsible for membrane insertion and pore formation [14]. Domain II consists of three anti-parallel β-sheets likely to be involved in receptor recognition [15] and the C-terminal domain III comprises of two antiparallel β-sheets arranged in a jelly roll-like topology [16] functions in ion channel regulation, receptor binding [17,18] and particularly in determining insect specificities [19-21]. This domain III region also possesses a remarkable similar topology to some carbohydrate-binding proteins suggesting that recognition by sugar molecule may be an important criterion for Cry toxin action [22]. 

To date, several Cry1Ac receptors have been identified, of which the best characterized are cadherin [23] and aminopeptidase N [24], after binding to which the actual intoxication process initiates. Apart from that alkaline phosphatase [25] and another 270 kDa glycoconjugate [26] had also been suggested as Cry toxin binding proteins. Although the mode of action of Cry toxin is not clearly understood, but till date it has been suggested that it is an intricate and multistep process involving sequential interaction with multiple receptors. After ingestion by the susceptible insects, the protease activated Cry1A toxin follows a “ping-pong” binding mechanism [27] in which the toxin monomer first bind to high abundant glycosylphosphatidylinositol (GPI) anchored alkaline phosphatase (ALP) or aminopeptidase N (APN) proteins as a mechanism to bring the toxins close to the insect midgut epithelium, followed by their interaction with cadherin protein that induces further cleavage of the helix α-1 region of domain I, leading to subsequent conformational change from monomer to oligomer [28,29]. These toxin oligomers again binds with high affinity to APN and ALP which are GPI anchored receptor located in specific membrane microdomain called lipid-rafts, leading to the membrane insertion by forming ion leakage pores that causes osmotic lysis, resulting in extensive damage to the midgut epithelial cells and eventual larval death [27,30,31]. Therefore, the overall action of Cry toxin, logically explains the absolute requirement of presence of specific receptors in the insect midgut and the main criteria for Cry toxin action primarily relies on the specific recognition of these receptors by toxin molecule. Binding of these toxins to their respective membrane receptors, which are preferentially associated with lipid rafts, promotes an increase in local toxin concentration within the cell membrane favouring toxin oligomerization required for pore formation, a crucial step in toxin action [32].

The Cry receptors characterized so far are mostly glycosylated proteins implying that carbohydrate residue plays an important role in toxin-receptor interaction and subsequent Cry toxin specificity [33]. In most cases, the interaction is mediated by the terminal N-acetylgalactosamine (GalNAc) moiety [34]. Earlier investigation of the Cry1Ac domain III region identified several amino acid residues that confer the sugar-binding property and in turn form the epitope [35]. Afterwards, studies on Cry1Ac-GalNAc co crystallization have shown that GalNAc binds in a unique cavity of domain III of Cry1Ac that further helped to identify directly the toxin residues responsible for recognizing the specificity determinant on insect APN [36]. Our previous study documented a membrane-bound ~138 kDa homodimeric alkaline phosphatase, HaALP that serves as potential receptor of Cry1Ac in an Indian isolate of *H. armigera* [37]. Lectin ligand blot confirmed that α-GalNAc residue at the non-reducing terminal of the glycan structure in the membrane bound HaALP protein mediates the toxin-receptor interaction. However, the identification of the key amino acid residues of Cry1Ac involved in HaALP receptor binding and the exact mechanism of interaction between GalNAc residues of the receptor towards domain III of Cry1Ac monomeric form remained elusive. Therefore, in the present study, we aimed to investigate the role of several domain III residues surrounding the GalNAc binding pocket in the Cry1Ac toxin -HaALP receptor interaction. Although it is well characterized that for membrane insertion and pore formation oligomeric form is important but in this study we have tried to understand the initial binding steps that occurred between Cry1Ac monomer and GalNAc containing HaALP. A mutagenesis approach was adopted and a series of alanine substitutions were made at Q509, N510, R511, Y513 and W545 position to facilitate the selective modification of the interactive residues and functional characterization of the mutants was carried out by biochemical, biophysical and computational analysis that suggested the importance of those residues in the proposed GalNAc-mediated Cry1Ac interaction and subsequent insect mortality. Analyses of the wild type (WT) and mutant toxin interaction towards the receptor by real time binding kinetics revealed a considerable understanding of the molecular basis of initial binding interaction between the Cry1Ac toxin monomer and HaALP receptor that has been discussed later.

## Materials and Methods

### Site directed mutagenesis

Site directed mutagenesis was performed using quickchange mutagenesis kit according to the manufacturer’s instruction (Quickchange kit, Stratagene, USA). The pQE-30 plasmid harbouring 1.8kb Cry1Ac DNA sequence was used as template. Altogether seven mutants have been generated by replacing Q509, N510, R511, Y513, W545, Q509-N510-R511 and Q509-N510-R511. Y513 with alanine. All the mutant plasmids were screened by DNA sequencing and positive clones were transformed into *E. coli* M15 cells. 

### Expression and purification of Cry1Ac and its mutants

Expression and purification of the WT and mutated Cry1Ac toxins were carried out following manufacturers’ instructions with some modification (Qiaexpressionist, Qiagen, Germany). His-tagged proteins were purified by metal-affinity chromatography with Ni-NTA column. Protein samples were analyzed in 10% SDS-PAGE [38] and subjected to Western blot analysis with anti-His antibody. 

### CD spectra analysis

Far UV CD spectra of Cry1Ac WT and mutant toxins were monitored in a Jasco spectropolarimeter equipped with a thermostatically controlled cell holder using a quartz cuvette of 1 cm pathlength. The proteins were diluted in 25 mM phosphate buffer (pH-7.5) to obtain 1.5 µM concentration and measurements were taken between 205 and 260 nm. All the samples were maintained at 25±10 °C and an average of nine scans were taken with a bandwidth of 5 nm. The final spectra were obtained by subtracting the buffer contribution from the original protein spectra. The CD results were expressed in terms of mean residual ellipticity (MRE) in deg.cm^2^ .dmol^-1^ and put in the following formula

$$\left[\theta \right]=\frac{{\left[\theta \right]}_{obs}}{10npcl}$$

Where θ_obs_ is the observed ellipticity in millidegres, n is the number of amino-acid residue, cp is the molarity, and l is the path length of the cell in concentration [39].

### Dissociation constant (K_d_) determination using fluorescence spectroscopy

Fluorescence spectra of WT and mutated toxins were measured in a spectrophotofluorometer (F-7000; Hitachi, Tokyo, Japan) equipped with a xenon lamp. WT protein (5 µM) was titrated with increased concentration of GalNAc and GlcNAc (control) from 5 to 100 µM in 25 mM Tris buffer (pH-8.0). In addition mutant protein samples (5 µM) were also titrated with GalNAc using the same incubation condition and measured in a Sigma cuvette (volume: 1 ml; path length: 1 cm). An excitation wavelength was set at 295 nm to selectively excite the Trp residues, and the emission spectra were recorded from 315-400 nm with the fixed slit width of 5 nm. The single-site ligand (GalNAc) binding equation measured through changes in the fluorescence intensity represented as

$$\frac{\Delta F}{C}=A-K_{a}\Delta F$$

where ΔF represents the increase or decrease in fluorescence intensity at a given concentration (C) of the ligand, K_a_ is the association constant, and A = K_a_ΔFmax where ΔFmax stands for the maximum change in fluorescence intensity [40]. The ΔF/C against ΔF was plotted and the slope (K_a_) was used to calculate the dissociation constant (K_d_) for binding of Cry1Ac to GalNAc. 

### Toxicity assay

Insect bioassay was conducted with *H. armigera* neonates (3-5 days old) by surface contamination method [41]. Artificial diet was prepared and poured into 24 well tissue culture plates. Cry1Ac WT and mutant proteins were diluted in 25 mM phosphate buffer (pH-7.2) and 40 µl of samples were applied per well (2 cm^2^) on artificial diet surface. One *H. armigera* neonate was placed in each well and kept undisturbed at 27±2 °C, 65±5% relative humidity, with a 16:8 hr light dark cycles. Five different concentrations (0-10 µg/ml) were used for each protein sample with 8 neonates per concentration. For negative controls insects were tested with same volume of buffer. Observations were recorded after 5 days for larval survival and larval weight. The entire assay was performed in triplicate and LC_50_ value for each protein was determined from the raw data by Probit analysis [42]. 

### Membrane bound Alkaline Phosphatase purification from *H. armigera* midgut

BBMV were isolated from second to third instar larvae of *H. armigera* provided by ICRISAT (Patancheru, India) following the magnesium precipitation method [43]. A total of 50 mg of BBMV samples were suspended in buffer containing 20 mM Tris-HCl (pH-7.4), 150 mM NaCl, 5 mM EDTA, 0.2 mM PMSF, 0.2% CHAPS, and incubated overnight at 4 °C. Insoluble materials were removed by centrifugation at 30,000 g for 30 minutes at 4 °C and supernatant was subjected to gel filtration chromatography as described previously [37]. After purification the fraction was resolved in 10% SDS-PAGE, two-dimensional gel electrophoresis and subsequent ligand blot experiment using Cry1Ac toxin. The protein spot detected in 2-D PAGE was analyzed by capillary liquid chromatography tandem mass spectrometry (LC-MS/ MS) [37] and identified as alkaline phosphatase receptor. 

### Ligand blot assay

According to the protocol described earlier [37] HaALP protein was resolved in 10% SDS-PAGE and electrophoretically transferred to Hybond C membrane (GE Healthcare, UK) in a Hoeffer (Hoefer Inc. Holliston, MA, USA) electroblot apparatus. The membrane was blocked with 5% non fat milk (Merck, Germany) in 1X PBS (pH-7.4) for 2 hours and incubated with 5 nM of Cry1Ac WT and mutant proteins in 1X PBS (pH-7.4) for 2 hours. The membrane was further washed with 1X PBS for 3 times and overlaid with Cry1Ac polyclonal antibody (1:3000 dilution) for 1 hr at 4 °C. After incubation the membrane was washed with 1X PBS (pH-7.4) as before and incubated with anti rabbit IgG HRP conjugate (1:20,000 dilution) (Sigma Aldrich, USA) for 1 hour. Finally the membrane was developed on Kodak X-ray film using an ECL kit (GE Healthcare, Germany). 

### Surface plasmon resonance

The interaction study between HaALP and Cry1Ac toxin was monitored through SPR analysis using a BioacoreX100 instrument and CM5 sensor chips (Biacore). The purified HaALP sample was concentrated through microcon device (Milipore) and subsequently diluted to 10µg/ml in 10 mM sodium acetate buffer (pH 5.5). The surface of CM5 chip was activated for 5 minutes at a flow rate of 10μl/ml by amine coupling method using a standard amine-coupling kit (Biacore). Short pulses of HaALP were injected across the activated surface until approximately 165 RU of HaALP was immobilized on flow cell 2. Following receptor immobilization this flow cell was blocked by a 5 minutes injection of 1 M ethanolamine at a flow rate of 10 μl/ml. HBS-N buffer (10 mM HEPES, pH 7.4, 150 mM NaCl) was used as both the running and sample buffer throughout the experiments. Purified Cry1Ac WT and mutant toxins were prepared in HBS-N buffer and then injected across this surface at a flow rate of 30 µl/min in five different concentrations. The complex was allowed to associate and dissociate and after each injection of analyte the ALP surface was regenerated with two 10-seconds injections of glycine-HCl, pH-2.0. Binding events were monitored in real time by global fitting of the data to 1:1 Langmuir binding model provided with the BIAEvaluation 3.1 software to determine the binding constant. Response curves were prepared by subtracting the signal generated simultaneously on the control flow cell.

### Homology modeling of Cry1Ac

Homology modeling of Cry1Ac was performed by CPH model 3.2 server [44] based on the X-ray crystallographic structure of Cry1Aa toxin from *B. thuringiensis kurstaki* strain HD1 (PDB accession code: 1CIYA) as the template structure with which it share 73% sequence similarity. The model was further verified with Ramachandran plot obtained from PROCHECK analysis [45] and quality of the structure was validated by ProSA [46] and ERRAT servers [47]. ProSA calculates the Z-score of the structure from the statistical analysis of the known protein structure while ERRAT score shows the overall quality factor for non-bonded atomic interactions.

### Molecular Docking

The docking of GalNAc into the carbohydrate-binding site of Cry1Ac was performed with AutoDock version 4.0 [48]. AutoDock 4.0 consists of two main programs: autodock – performs docking of the ligand to a set of grid that describes rigid target protein and autogrid – pre-calculate these grids. Graphical user interface of AutoDock, AutoDockTools (ADT), built on Python Molecular Viewer was used for Docking. After homology modeling energy minimization of the validated Cry1Ac structure was performed with ABNR using CHARMM22 force field [49] for 5000 steps to remove any bad contacts in the starting model of the molecule and selected as receptor for docking. Hydrogen atoms were added to the modelled structure and converted into PDBQT format by AutoDock. The starting coordinates of the ligand (GalNAc) was prepared by sketching the molecule using Discovery Studio 3.1 Visualizer and its geometry was optimized using fast Dreiding-like forcefield where six rotatable bonds were found. A 3D grid box of size 60 x 62 x 54 was defined, with a grid space of 0.375 Å that covered the above mentioned residue. The standard docking protocol was performed using Lamarckian genetic algorithm by keeping receptor as rigid and ligand as flexible. Altogether 10 autodock runs were performed using an initial population of 150 randomly placed individuals, a maximum number of 2500000 energy evaluations, a mutation rate of 0.02, a crossover rate of 0.8 and elitism rate of 1. Results were clustered according to the RMSD tolerance value of 2 Å and binding free energy and inhibition constant was calculated for each AutoDock run. 

### Molecular Dynamics Simulation

After docking the best conformation was selected based on the estimated binding energy and molecular dynamics (MD) simulation was performed. To understand the effect of mutation of specific residues on GalNAc binding, seven systems were prepared and MD simulation was performed. Each system was dissolved in the TIP3P water box ensuring the minimum thickness of at least 9 Å everywhere. Counter-ions were added to neutralize the system. Prior to MD simulation each system was minimized using 1000 steps of ABNR followed by NAMD for 2000 steps then heated upto300K and then equilibrated for 30 ps. Van der Waals interactions were truncated at 12 Å and particle mesh ewald (PME) [50] was used to calculate the long range electrostatic interaction. No constraint on the bond was imposed. NAMD requires x-plor psf, which was generated by c35b6version of CHARMM package. CHARMM22 force field was used to represent the protein and the charmm generalized parameter (CGENFF) [51] was used to represent the GalNAc. Ten trajectories each of one nanosecond was saved in the production run. The movies were prepared using VMD [52] and molecular figures were prepared using Pymol [53]. To get an idea about the impact of specific mutation on the other residues, solvent accessible surface area (SASA) has been calculated over the last frame using naccess.v 2.1.1 [54]. Interaction energy for each of Q509, N510, R511, Y513 and W545 with GalNAc was calculated for ten nanoseconds simulation of WT. The binding energies (*ΔE*_*binding*_) were obtained from the interactions between the ligand and the WT and mutant of Cry1Ac protein compelxes. The change of the binding energies (*ΔΔE*_*binding*_) was calculated by taking the difference of *ΔEmut* from the same value of WT (*ΔEwt*). 

## Results

### Expression and purification of WT and mutant toxins

**Figure 1:**
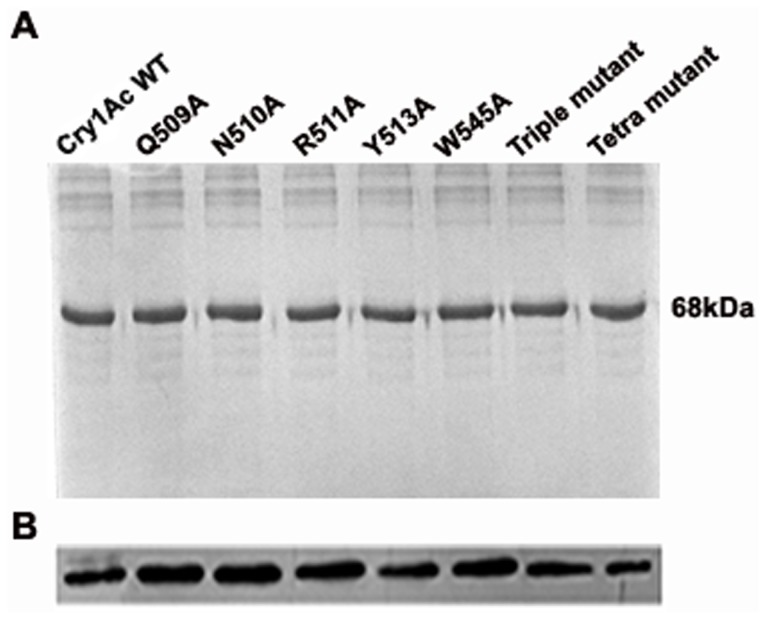
SDS-PAGE and western blot analysis of WT and mutant proteins. (A) His-tagged proteins were purified by metal-affinity chromatography and fraction containing Cry1Ac proteins resolved in 10% SDS-PAGE. 68 kDa represents the apparent molecular weight of the protein. (B) Protein samples were electrophoretically transferred to nitrocellulose membrane and detected with anti-His antibody that shows a clear band corresponding to the size of Cry1Ac toxin.

**Figure 2:**
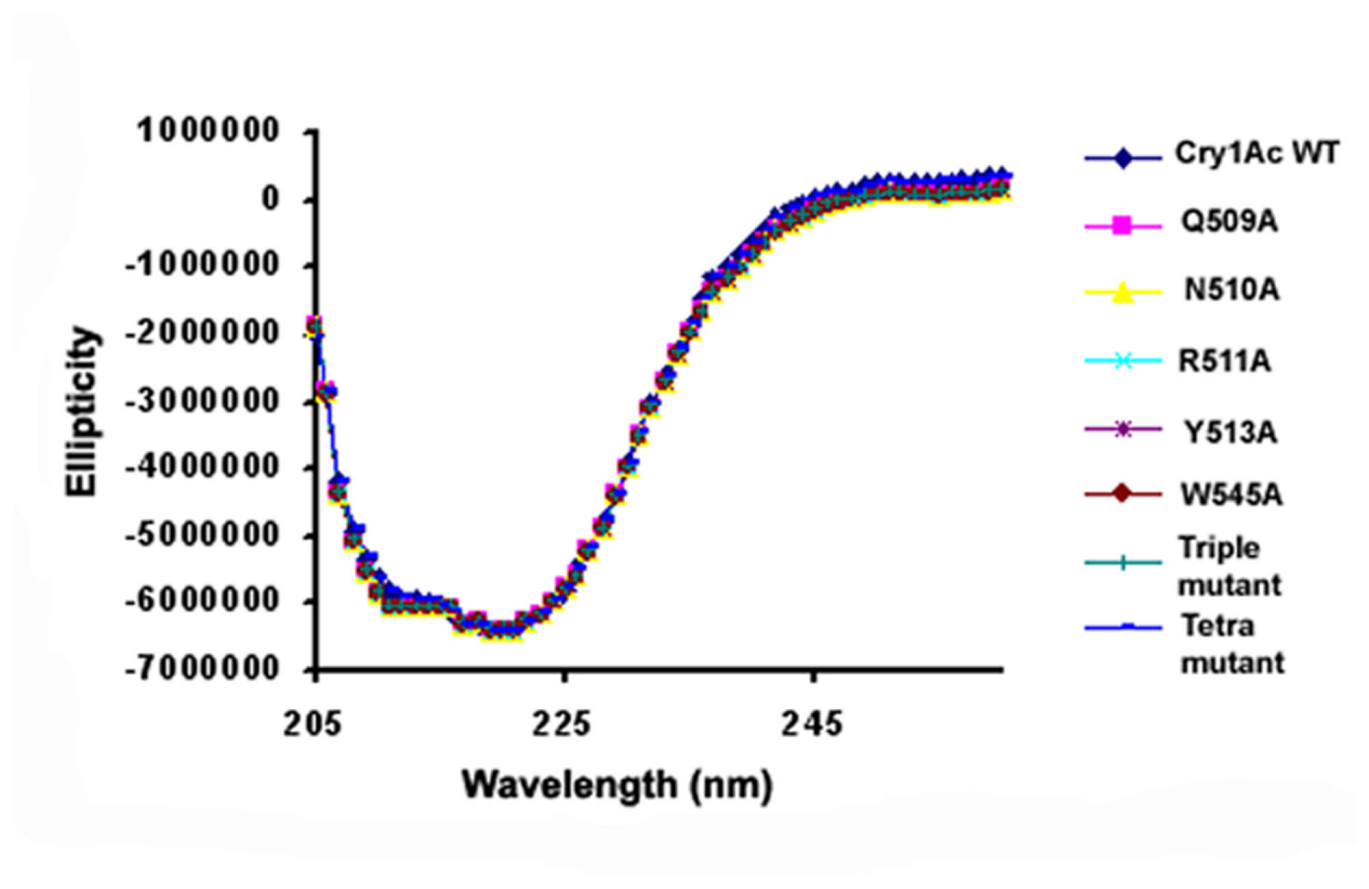
Far-UV CD spectra of Cry1Ac WT and mutants. It shows overlapping spectra for all the proteins suggesting that mutations of the selected residues did not induce any further structural changes in toxin conformation.

Cry1Ac WT and mutant toxin expression was carried out in *E. coli* M15 cell line that produced soluble toxin monomer at approximately 68 kDa region of 10% SDS-PAGE (Figure 1A) detected with Western blot analysis with anti-His antibody (Figure 1B). DNA sequencing method confirmed the identity of the individual toxin. To analyze possible structural changes imposed by mutation, CD and fluorescence spectroscopy was performed that showed identical spectrum suggesting that mutations of the selected residues did not induce any structural changes in toxin conformation (Figure 2).

### Determination of K_d_ values using fluorescence quenching

**Figure 3:**
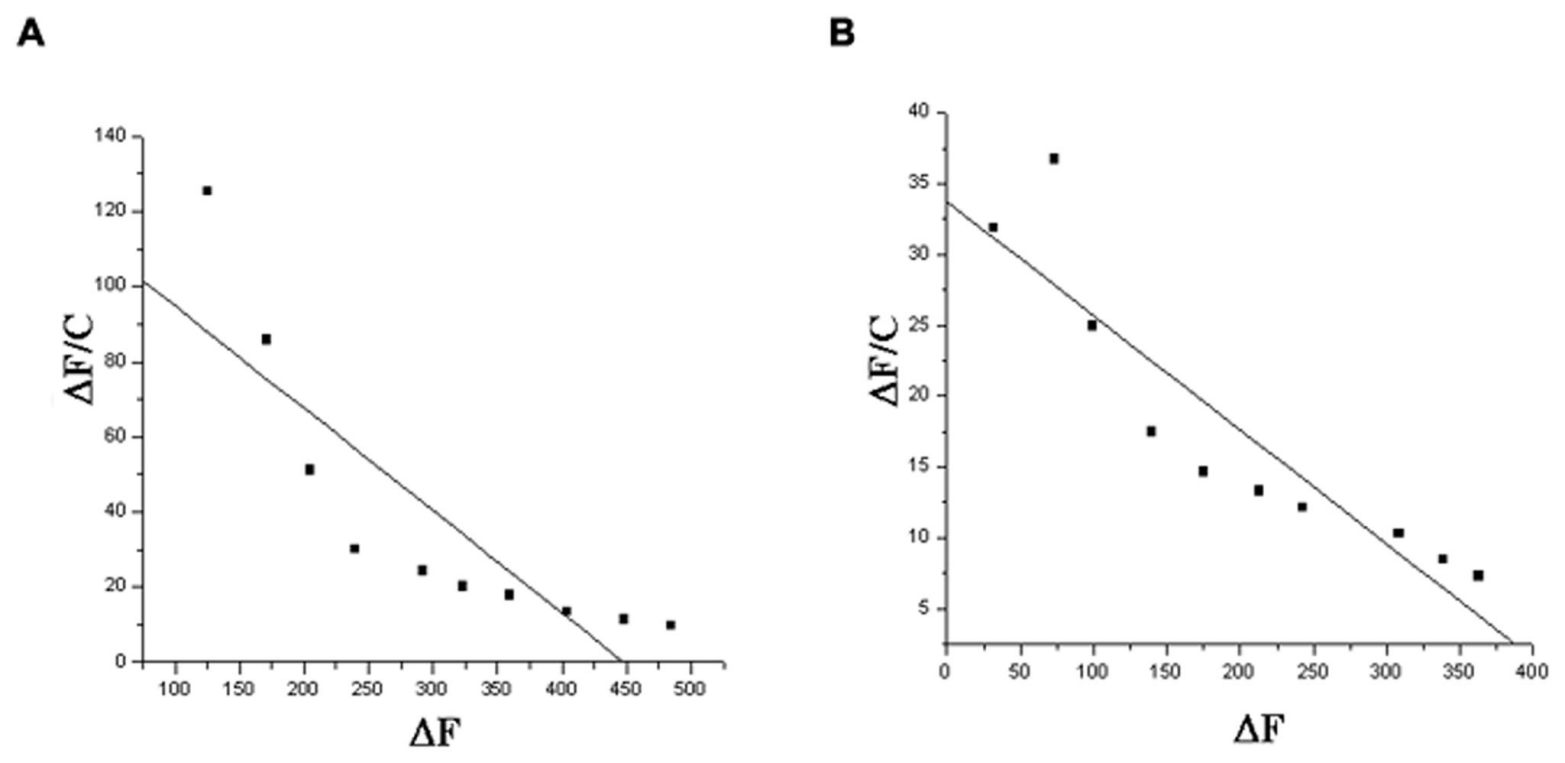
Determination of K_d_ value from fluorescence quenching method. The ΔF/C against ΔF was plotted and the slope (K_a_) was used to calculate the dissociation constant (K_d_) for binding of Cry1Ac to GalNAc. (A) WT Cry1Ac (B) Tetra mutant.

Fluorescence emission spectra were taken for all the proteins that exhibited identical profile with λ_max_ at 333 - 335 nm (Figure S1). Upon GalNAc addition the obtained K_d_ value of WT toxin was found to be 3.67 µM whereas, with GlcNAc addition it showed K_d_ value of approximately 27.77 µM. (Figure S2). A similar pattern of quenching was detected for most of the mutants with GalNAc; however, the tetra mutant (Q509A-N510A-R511A . Y513A) showed a very small change in fluorescence intensity. The calculated K_d_ values are shown in Table 1, and representative plots are shown in Figure 3 A, B.

**Table 1:** Determination of binding constant (K_d_) of Cry1Ac WT and tetra mutant from fluorescence titration method with GalNAc.

Proteins	K_d_
Cry1Ac WT	3.67 µM
Tetra mutant	12.50 µM

### Effects of mutation on insecticidal activity

**Figure 4:**
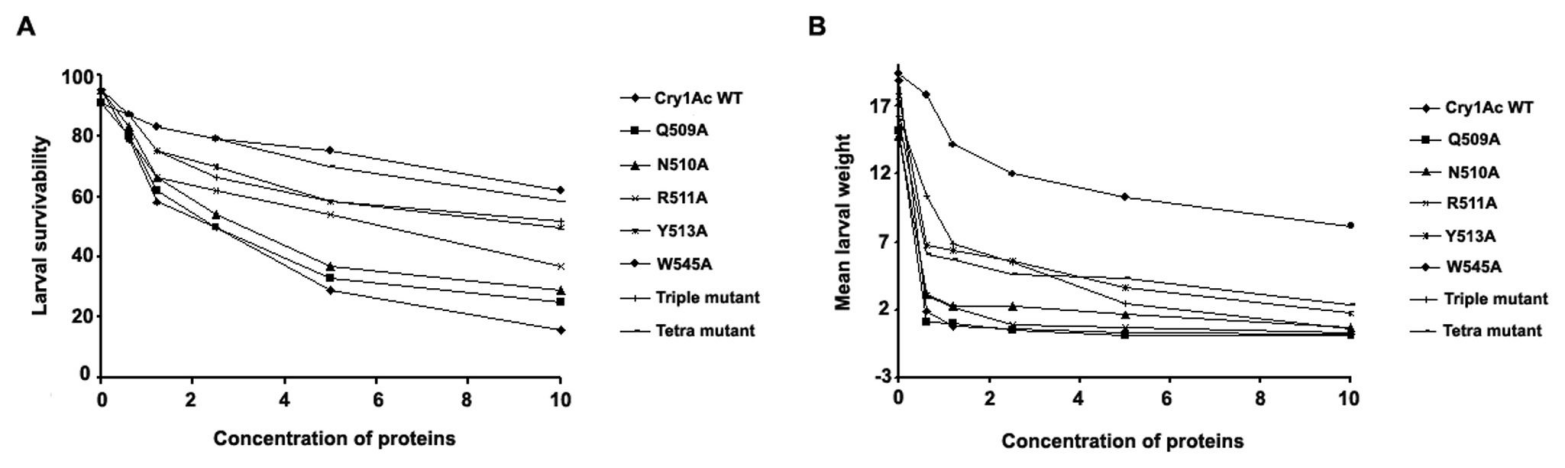
Insecticidal activity of WT and mutant proteins against *H. armigera*. WT and mutant protein samples were applied on artificial diet surface and one *H. armigera* neonate was released in each well. The plates were left undisturbed for 5 days at 27±2 °C, 65±5% relative humidity, with a 16:8 hr light dark cycles and observations were recorded after 5 days. X-axis represents concentration of proteins in µg/ml and Y-axis represents percentage of larval survival (A) and mean larval weight (mg) (B).

To determine the mutational effects of individual toxins on larval toxicity *H. armigera* bioassays were conducted. After 5 DAI (days after infestation) the larval survival rate and the mean larval weight (mg) were monitored and compared (Figure 4 A, B). The LC_50_ value was obtained using a Probit analysis that ranged from 2.34 to 34.15 µg/ml of protein (Table 2), indicating a 15- fold variability in the different mutant forms. The WT Cry1Ac toxin conferred highest toxicity against the susceptible larvae with a 16% survival rate and a mean body weight of 0.3 mg, whereas R511A and Y513A mutants were found to be 3- and 4-fold less toxic than the WT. In contrast to this, Q509A and N510A mutants exhibited almost similar level of larval survivability as the WT toxin, showing that these two mutants maintained larval toxicity even after mutation. Triple and tetra mutants have generated a significantly reduced toxicity, implied that these residues have a combined effect on insecticidal activity and are important for maintaining toxicity. Interestingly, mutant W545A, being a point mutation found to be least effective in its insecticidal activity compared to all the other mutants. The changes in larval mean body weight were also monitored as it can also be a consequence of toxin action and differences in larval weight was obtained. 

**Table 2:** Biological activities of Cry1Ac toxins and their mutants on first instar larvae of *H. armigera* treated on artificial diet.

Cry1Ac proteins	LC_50_ value (µg/ml)	Fiducial limit of LC_50_		SE slope	Regression equation (Y)	Χ^2^ value	df
		Lower 95%	Upper 95%				
Cry1Ac WT	2.35	1.82	2.99	0.323	4.44 ± 1.49x	0.42	3
Q509A	3.13	2.44	4.05	0.340	4.32 ± 1.36x	0.33	3
N510A	3.49	2.69	4.70	0.319	4.28 ± 1.31x	0.39	3
R511A	5.95	4.05	11.17	0.309	4.28 ± 0.91x	0.37	3
Y513A	11.49	7.11	31.66	0.399	3.81 ± 1.10x	0.42	3
W545A	34.15	19.70	93.61	0.503	3.50 ± 0.97x	0.10	3
Triple mutant	9.95	6.21	24.10	0.332	4.03 ± 0.97x	0.39	3
Tetra mutant	22.57	14.69	44.67	0.374	3.76 ± 0.91x	0.11	3

24 larvae were measured per concentration. Results were observed after 5 days of infestation of toxins and calculated as LC_50_ using Probit analysis.

### Effect of mutation on receptor binding

**Figure 5:**
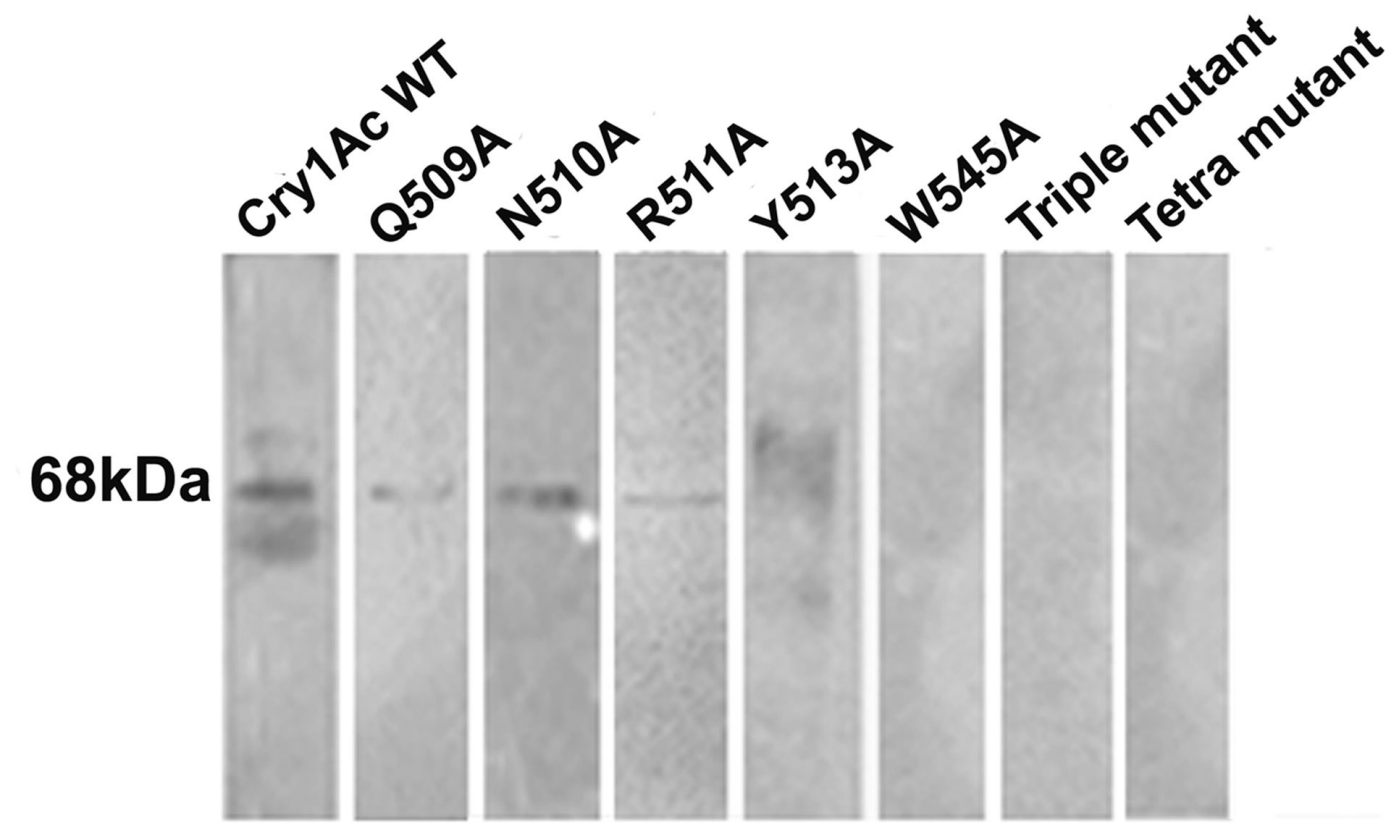
Cry1Ac WT and mutant toxins binding to *H. armigera* ALP. HaALP receptor protein was resolved by 10% SDS–PAGE and transferred to nitrocellulose membrane. Each lane containing 2 µg of BBMV protein was treated with 5 nm of Cry1Ac WT and mutant toxins. The major Cry1Ac binding protein is indicated by position of the band at approximately 68 kDa position.

Cry1Ac WT and mutant toxin binding to purified HaALP receptor was monitored using ligand blot analysis. WT Cry1Ac showed a major band at ~68 kDa (Figure 5) for the receptor interaction. Despite the mutations, receptor-binding was detected for Q509A, N510A and Y513A mutants. The binding ability of R511A and W545A mutants was substantially reduced whereas binding was almost abolished for the triple and tetra mutants.

### Estimation of binding kinetics using SPR

**Figure 6:**
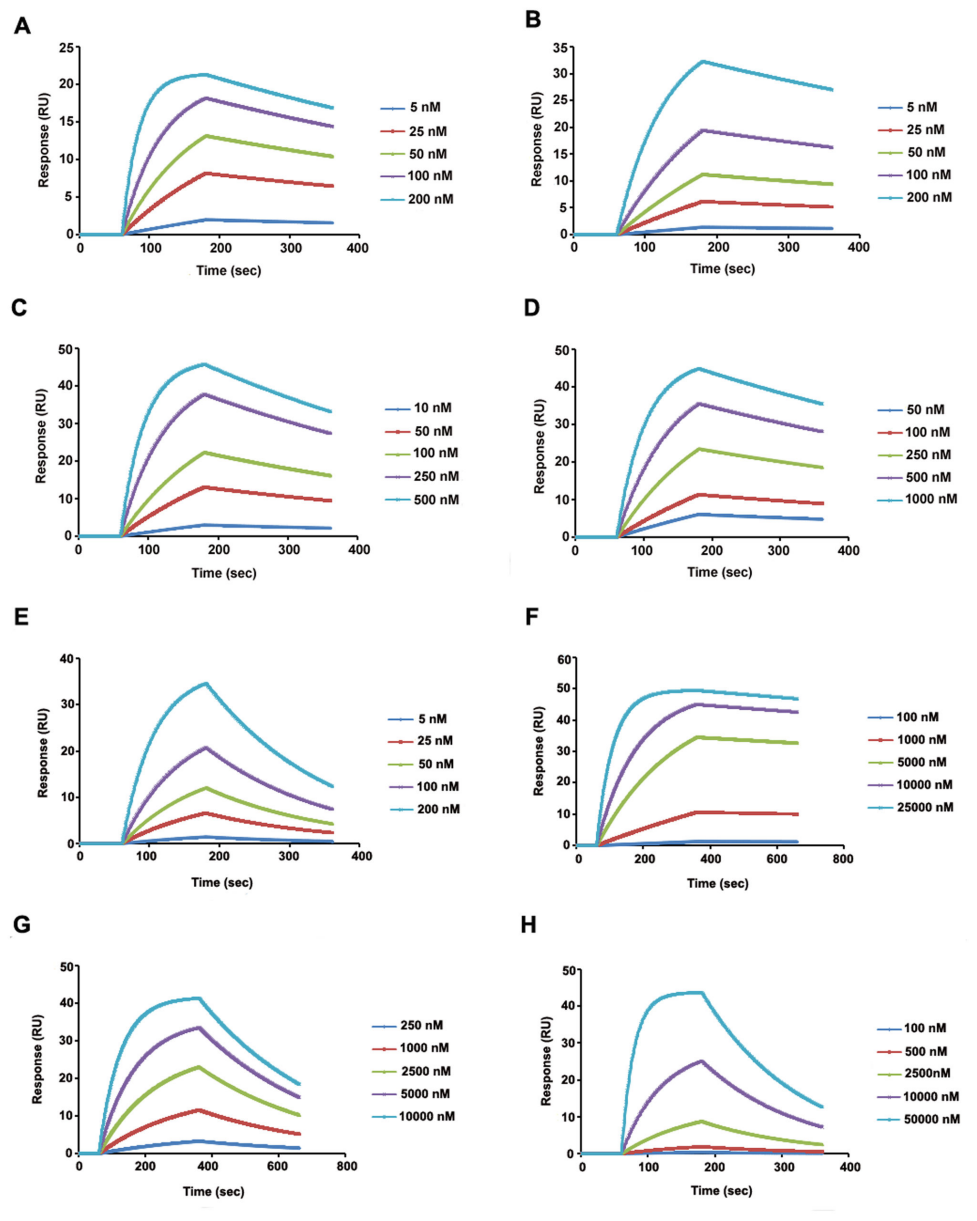
Sensorgrams of Cry1Ac binding. The purified HaALP sample was immobilized on CM5 surface and five different concentrations of WT and mutant toxins were injected at a flow rate of 30 µl/min. Binding events were monitored and response curves were prepared by subtracting the signal generated simultaneously on the control flow cell. (A) Cry1Ac WT, (B) Q509A, (C) N510A, (D) R511A, (E) Y513A, (F) Triple mutant, (G) Tetra mutant, (H) W545A.

SPR was used to study the real-time binding kinetics of the toxin-receptor interactive event. Response curves with various analyte concentrations were obtained, and the formation and decomposition of toxin-receptor complex was monitored. Using the time dependant kinetic data, the association (Ka) and dissociation (Kd) rate constants and the equilibrium binding constants (KD, KD=Kd/Ka) were calculated (Table 3). The kinetic study was performed for all the mutants, and dose dependency was observed (Figure 6, A-H). WT Cry1Ac had the highest affinity for HaALP of 7.6 nM, which was calculated from the observed Ka and Kd values. The KD values obtained for the Q509A and R511A mutant were 3- to 4- fold lower respectively than WT toxin. A significant decrease in affinity was observed for the N510A mutant. The Y513A and triple mutant had similar affinities, which were approximately 10- fold lower than the WT. In case of the tetra mutant minimum affinity was observed for HaALP while W545A mutant showed 3- fold lower affinities than that of tetra mutant.

**Table 3:** Binding kinetics of Cry1Ac WT and mutant toxins to HaALP receptor.

Toxins	Ka (1/Ms)	Kd (1/s)	KD (M)	Chi^2^
Cry1Ac WT	1.680E+5	12.91E-4	7.681E-9	0.52
Q509A	19.31E+4	0.005710	2.956E-8	11.4
N510A	2.32E+4	0.001355	5.838E-8	1.65
R511A	5.58E+4	0.001788	3.200E-8	11.6
Y513A	6.61E+3	0.001469	2.229E-7	3.60
Triple mutant	10.25E+3	0.002687	2.624E-7	12.7
Tetra mutant	0.75E+2	1.837E-4	2.424E-6	16
W545A	9.63E+2	0.006882	7.146E-6	0.353

Ka = Association rate constant

Kd = Dissociation rate constant

KD = Apparent affinity (Kd/Ka)

### Molecular insights of GalNAc binding

**Figure 7:**
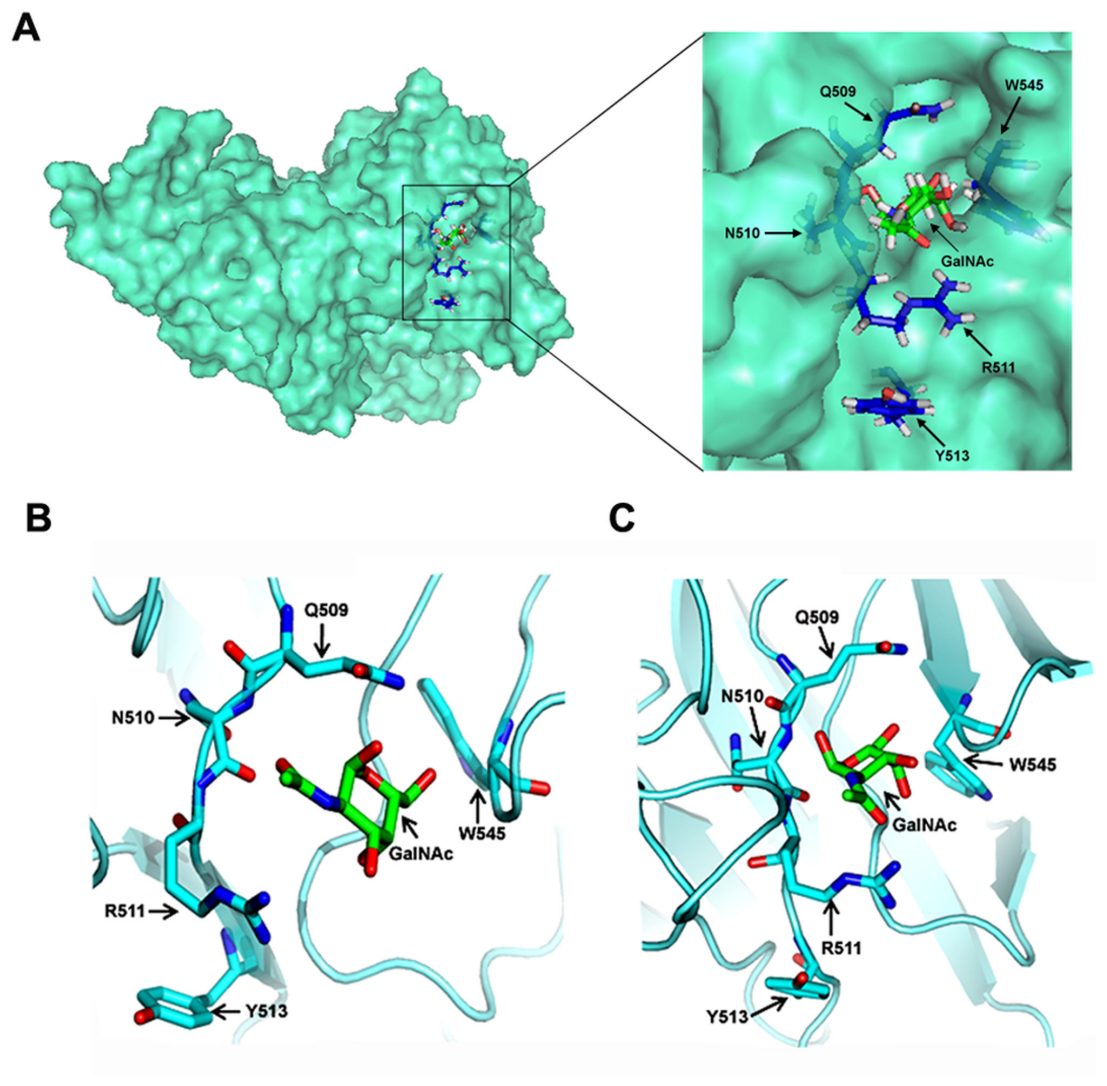
Docking of GalNAc into the homology model of Cry1Ac. (A) Surface representation of the Cry1Ac protein showing GalNAc binding pocket. Mutagenesis sites around the GalNac binding pocket are shown in stick conformation. Inset showing a close up view on binding site. (B) Before simulation GalNAc lies within the binding pocket and (C) after simulation GalNAc relaxing in the pocket to attain a cozier fit.

By means of automated docking, multiple conformations of the protein-ligand complexes were obtained from which the most preferred conformation (Figure 7A) was selected on the basis of estimated binding energy (Table S1). Root mean square deviation (RMSD) of backbone atoms and root mean square fluctuations (RMSF) of all heavy atoms of the WT Cry1Ac and mutants were calculated according to the trajectories. RMSD and RMSF values show that each simulation reached stable condition within the 10 ns timescale (Figure S3, A-B). 

In case of WT Cry1Ac-GalNAc complex, the selected docked structure shows that, the GalNAc molecule sits within a cavity on the protein surface (Figure 7B) and forms contacts with Q509, R511, N544, N547, N585 and V587 (Figure S4). During the 10 ns MD simulations, the system became more relaxed by optimizing interactions at the protein-ligand interface and H-bonds were formed between pairs of residues at the vicinity of ligand which help to hold the ligand tightly within the pocket throughout the run (Movie S1). As the time increases, the binding pocket achieves a 'cozier' fit as the side chains reorient themselves to grip the ligand well and the ligand remains associated within the protein cavity (Figure 7C). However, in case of tetra-mutant, lack of proper interactions among the residue side chains leads to a loosening of the pocket, and the ligand dissociates out from the pocket (Figure S5, A-B, Movie S2). 

**Figure 8:**
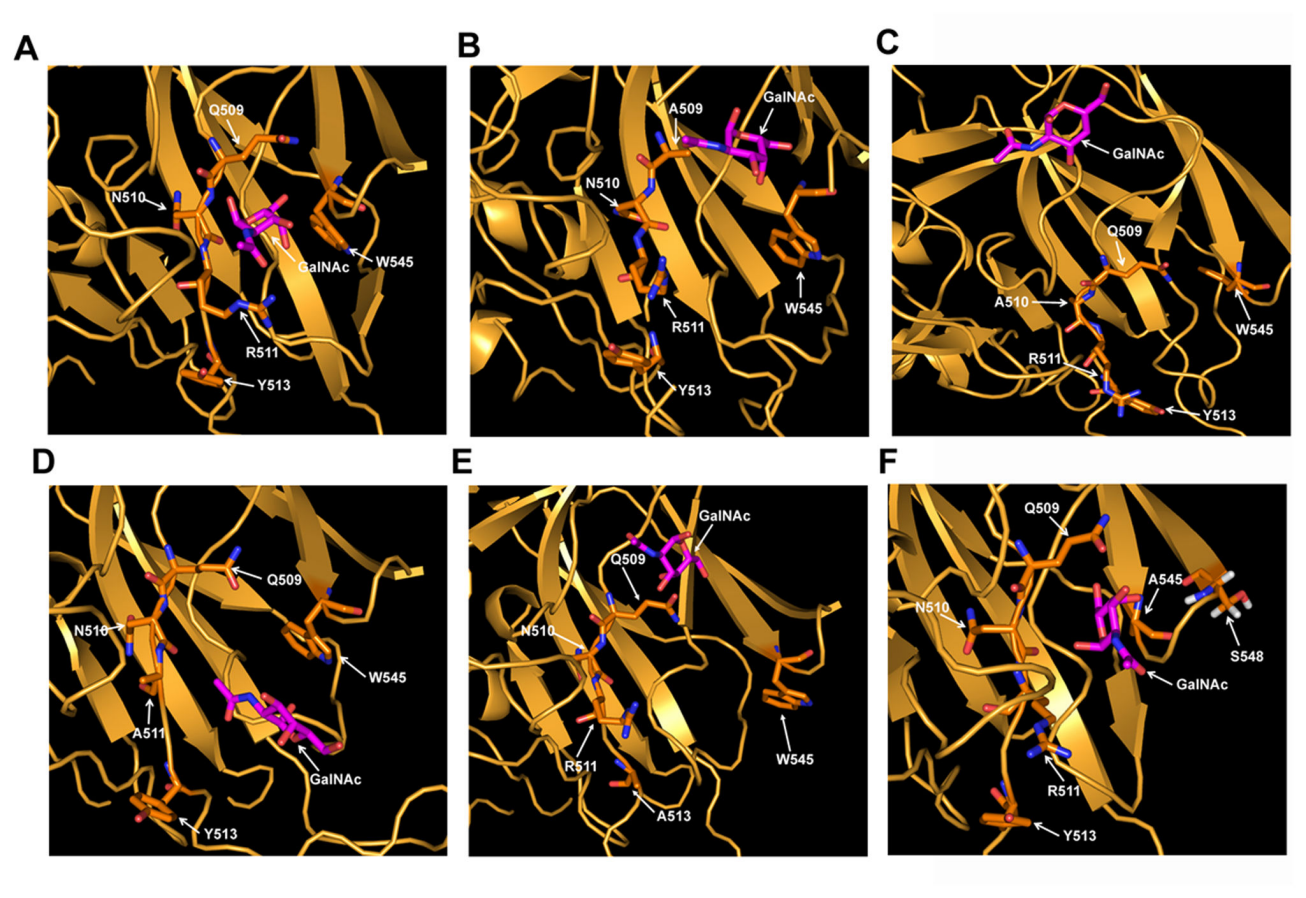
Comparison of the GalNAc binding modes of Cry1Ac and its mutants. Snapshots were taken during MD simulation of Cry1Ac-GalNAc interaction. (A) GalNAc binding with WT Cry1Ac. (B) Outward movement of GalNAc after Q509A mutation. (C) Replacement of Asn with Ala leads to significant change in GalNAc orientation. GalNAc moved far away from the binding pocket due to the mutation. (D) GalNAc remained in the binding pocket. (E) Y513A mutation leads to the opening of pocket and GalNAc moved away. (F) GalNAc moves closer to A545 while losing its contact with Q509, N510 and R511. Binding pocket is opening due to loss of bulky side chain of Trp residue that made it most suitable for maintaining the integrity of the binding cleft.

To unravel the contributions of single amino acid residue in the binding cleft for which the pocket becomes fragile, individual simulation of each mutated structures were run. No drastic change was observed for Q509A or R511A except for the disruption of H-bonds associated with the orientation of the ligand (Figure 8 B, D). The most disruptive changes were observed for N510A and Y513A residues, where the ligand moved away from the pocket (Figure 8 C, E) (Movie S3 and Movie S4). Apart from that, an interesting observation was made for the W545A mutant, where the replacement of the hydrophobic residue showed disruption in the integrity of the GalNAc binding site in domain III. The mutated W545A residue interacts with S548, and ligand moved closer to A545 by disrupting the H-bonds with R511 and Q509 (Figure 8F). Moreover, it genuinely shows us that mutation of this residue leads to the loss of compactness in GalNAc binding pocket due to the loss of packing interactions. In addition to this, due to short side chain of alanine, the mutated residue become unsuitable for maintaining the integrity of the binding cleft, which in turn proves this residue as a vital one for receptor interaction.

**Figure 9:**
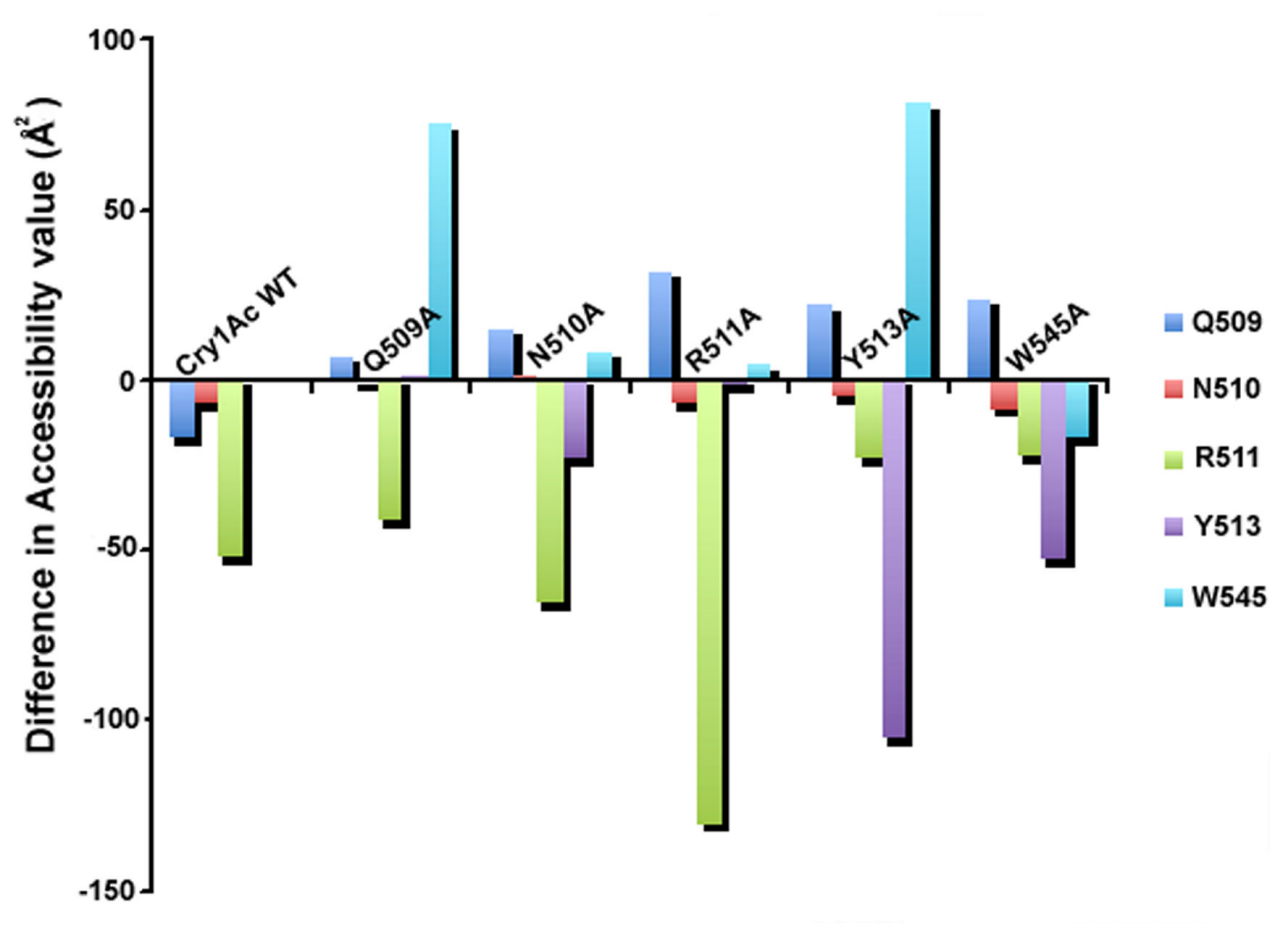
Analysis of solvent accessible surface area. It depicts effect of each mutated amino acid residue over the other residues. X-axis represents simulation of Cry1Ac WT and mutant residues. Y-axis represents accessibility value (Å^2^).

To understand the effect of each residue in binding cleft to fit the GalNAc during interaction the solvent accessible surface area (SASA) was calculated with the presence and absence of GalNAc molecule over the last frame. It points out interesting observations. Again it shows individual residue Q509, N510, R511 and Y513 each is important because due to Ala mutation Q509A is deflecting W545 which maintains the integrity of the cleft, N510A is deflecting both Q509 and W545, and R511A is deflecting mostly Q509, and Y513A is deflecting both Q509 and W545 a lot (Figure 9). This entire SASA calculation gives us the realistic picture where the effects of each amino acids in the GalNAc binding cleft has been justified by the Ala substitution and consecutive GalNAc binding simulation.

To obtain a gross idea of the ligand binding energies, interaction energy (i.e. Van der Waals and electrostatic interactions) of ligand for Q509, N510, R511, Y513 and W545 residues of Cry1Ac has been calculated. These calculations provide an estimate of the binding modes and affinities of the WT and mutants towards the ligand. During 10ns simulation of WT, the maximum interaction was observed with R511 and Q509 and insignificant contribution was obtained in other 3 cases (Table S2). While comparing the difference of binding energy (*ΔΔE*_*binding*_) of WT with every single mutant, interesting observation was found (Table 4) which showed drastic increase of binding energy of about 15- fold for mutating N510 to Ala after the simulation run. Similarly, a relatively comparable trend was observed in *ΔΔE*_*binding*_ calculation in case of Y513A and W545A mutations as their effects were not negligible but quite important for ligand interaction. This entire study shows that although Y513 and W545 do not directly affect the binding but in combination with Q509, N510 and R511, they strengthen the binding with GalNAc.

**Table 4:** Average interaction energy calculated over fourth trajectory between ligand and receptor for single mutants.

Name of system	*ΔEmut*	*ΔΔE*_*binding*_= *ΔEmut*-*ΔEwt*
Q509A	-28.77	16.57
N510A	-3.60	41.74
R511A	-16.40	28.95
Y513A	-32.58	12.77
W545A	-35.86	9.49

*ΔEwt*= - 45 35 Kcal/mol.

## Discussion

Over the years, *B. thuringiensis* coded Cry1Ac toxin has been established as a potent insect control agent. Although the widespread use of different Cry proteins in agriculture provided an enormous long-term selective pressure, the emergence of resistant insects threatens the effectiveness of these toxins. This problem necessitated the identification and development of modified versions of Cry toxin that might have broader insect specificities. But before that, a more precise and comprehensive investigation of the toxin receptor interaction is needed by elucidating the molecular insights of the epitopes of toxin molecule in binding which is a prerequisite for developing a reasonable understanding of the mechanism of action of each Cry toxins toward target insects. Previous studies have reported cadherin and APN type receptors and identified the specific epitopes that mediate the Cry1Ac binding characteristics in *H. armigera* [55,56]. It is now well established that some regions of Cry1Ac can interact with the terminal GalNAc residue of different receptors to mediate the toxin-receptor interaction, but it is not known yet whether the GalNAc residue in the terminal side chains of different receptors are interacting similarly or not. It is also unclear how the glycosylation chains are packed on different three-dimensional structures of the receptors. Nevertheless, virtually no information is available for the HaALP receptor binding determinant in Cry1Ac molecule and about the dynamics of the interaction in the binding cleft.

Alanine substitutions in the domain III of Cry1Ac toxin helped us to elucidate the selective binding of several residues to GalNAc moiety. Terminal GalNAc has been found in several different types of receptors of Cry1Ac, so the residues located in the GalNAc binding cleft are significantly important to maintain the interaction and their modification alters the ability to bind to the receptor in GalNAc mediated interaction. In the current study, we evaluated the role of several important domain III residues of Cry1Ac, Q509, N510, R511, Y513, and W545 that play important roles in sugar mediated receptor recognition. The mutational strategy helped us to study the comparative insecticidal activities and binding properties of the WT and mutants. Molecular dynamics simulation studies of the WT and mutant Cry1Ac proteins with GalNAc were also conducted to probe the structural effects of these mutations on GalNAc selectivity. The functional studies along with computational analysis provided a more transparent picture for evaluating the initial binding mechanism of Cry1Ac monomer and HaALP receptor interaction. 

The 1.8 kb *cry1Ac* WT gene sequence was used as template for mutagenesis and WT and mutant proteins were expressed in *E. coli*. Soluble proteins of approximately 68 kDa were purified and subjected to CD and fluorescence spectroscopy. The results showed that mutagenesis causes minimal perturbations in the folding patterns of the various mutants but causes significant differences in binding to HaALP receptor and toxicity toward target insect larvae. To determine the sugar specificity of WT and mutant Cry1Ac for the GalNAc molecule fluorescence quenching analysis was performed. A 4- fold difference in the K_d_ value was observed for the tetra mutant, which indeed displayed a reduced affinity for the GalNAc molecule. In case of WT, approximately 9- fold decreased affinity was observed for GlcNAc as compared to GalNAc therefore; further interactional studies involving GlcNAc with other mutants have not been considered.

Functional characterizations of the WT and the mutant proteins were studied by determining the binding constant and lethal doses necessary for insect mortality that provided evidence of functional epitopes on Cry1Ac domain III. Each residue contributed in binding in its own way. In bioassay experiment considerable differences in toxicity between WT and mutant toxins were observed. Y513A, W545A, triple and tetra mutants were found to be incapable of exhibiting significant toxicity, whereas mutant Q509A, N510A and R511A showed only 1.5 - 3 fold decreased toxicity than WT. Similar trend was observed in ligand blot analysis also where, W545A, Y513A, Q509A-N510A-R511A and Q509A-N510A-R511A.Y513A mutants did not show any significant binding but Q509A, N510A and R511A residue showed low binding affinity. Previous studies by Lee et al, have shown that alanine substitution mutations at the residues Q509, R511, and Y513 in the domain III of Cry1Ac toxin affected toxicity and binding to *Manduca sexta*, *Lymantria dispar*, and *Heliothis virescens* BBMV [57]. 

Therefore, to determine the real time binding kinetics of these proteins with the HaALP receptor SPR analysis was performed. The obtained affinity towards HaALP was found to be 3 orders of magnitude greater than the observed affinity towards GalNAc molecule obtained through fluorescence study. Presumably, the initial recognition is made through lectin like domain III region with GalNAc molecule but receptor-binding epitopes localized to specific residues in domain II region also play an important role in binding. During SPR analysis as both the domains take part, the GalNAc independent binding cannot be avoided. 

WT toxin showed higher affinity (7.6 nM) towards receptor than previously reported literature [58,59], possibly due to presence of membrane associated glycolipids in the present HaALP sample. Previously authors have expressed alkaline phosphatase from different insects using bacterial expression system [60], and some have experienced lower affinity binding towards receptor due to absence of glycosylation [61]. During BBMV preparation in CHAPS buffer although the GPI anchored portion gets removed by endogenous phospholipase treatment [62] but it has been experienced that some neutral lipids remain adhered with the protein [63]. These lipid aggregates further help in toxin insertion into lipid monolayer or bilayer [64] that forms cation and anion channels into lipid layer [65,66]. Moreover, the adherence of neutral lipids with receptor molecule would be advantageous [63] for toxin-receptor interaction. Presumably, in the present study during HaALP purification some neutral lipids remained associated with receptor molecule may have enhanced Cry1Ac toxin binding, as it is already known that glyocolipids also act as Cry toxin receptor [67]. 

Among the mutants, the binding and toxicity properties of Q509A and R511A were indistinguishable from that of WT; indicate that these residues are insignificant in receptor interaction. Mutant Y513A with 29- fold decrease in binding affinity and 4-5 fold lower toxicity compared to WT; supported the previous observation that Y513A residue of Cry1Ac caused a decrease in relative toxicity and a decreased APN-binding ability [57]. 

Although, W545A exhibited only 1.5 fold differences in K_d_ value (5.57 µM) during fluorescence study (Figure S6) compared to WT toxin but showed a significant differences in toxicity and binding detected by SPR analysis. The similar discrepancies were obtained in case of N510A mutant also where mutation leads to only 1.6 fold differences in K_d_ value (6.09 µM) as compared to WT (Figure S7). In case of fluorescence quenching study, fluorescence intensity was measured with the addition of GalNAc molecule. Whereas, in SPR, purified HaALP was immobilized on the surface. Hence, the magnitude of observed changes in K_d_ values obtained from fluorescence study, may not be comparable to the KD values detected by SPR. The accuracy of the kinetic measurements performed through SPR analysis helped us to understand more intensely the interaction between toxin and HaALP receptor. To understand the molecular phenomena, homology modeling followed by a large-scale MD simulation was performed. The developed homology model showed overall good structural quality which was confirmed using several different validation tools. Molecular docking was performed with the GalNAc and overall stability of the complex was investigated using MD simulation. To know the mode of ligand binding, seven sets of different MD simulation were run and significant change from the initial docked structure was observed. As the ligand reoriented itself to maximize its contacts, the complementary changes in the orientations of the side chains around the binding pocket were noted. In the WT Cry1Ac-GalNAc complex, the orientation of the GalNAc changed slightly within the groove but was not expelled from the binding pocket. In contrast, the GalNAc moiety behaved differently in mutant toxins and showed reduced affinities for mutated residues. In case of W545A mutation, it shows an indirect effect in GalNAc binding that contributes to approximately 9 Kcal loss of stability of the complex. In the WT complex W545 residue actually helps to maintain the GalNAc binding pocket where the bulky side chain of the Trp residue makes it suitable for maintaining the integrity of the binding cleft, and a mutation in this residue reduces the compactness of the binding pocket due to the loss of packing interactions. This feature appears to be a mechanism for keeping the ligand in a preferable and functional orientation for the interaction to happen. It is suggested that the maximum decrease in binding affinity in SPR analysis has been reflected in the bioassay that finally leads to maximum decrease in insecticidal activity in case of W545A mutant. Therefore, both SPR and insect bioassay data revealed the importance of this residue in determining GalNAc-mediated receptor specificity. Presumably, the less efficient binding of W545A mutant during initial course of action may have reflected in oligomer mediated further binding which ultimately lead to decreased toxicity. 

Mutant N510A with 8- fold decreased binding affinity exhibited only 1.5- fold lower toxicity towards *H. armigera* neonates as compared to WT. This result indicates to the fact that SPR examines direct interaction between toxin and purified receptor whereas, for exerting toxicity several other factors are involved. Relationship between toxicity and purified receptor binding does not always correlate. During bioassay whole midgut becomes exposed to toxin and as toxin oligomerization occurs, subsequent interaction come into the picture that ultimately leads to insect mortality. The present study highlights to understand the very initial interaction between Cry1Ac monomer and HaALP receptor and we have considered only the domain III mutants that are affected in GalNAc mediated receptor binding, however, during bioassay the involvement of other receptors (GalNAc independent) cannot be ignored. So, binding with one particular receptor may not be proportionally correlated with the overall toxicity. Similarly, when we considered N510A mutant binding to total BBMV proteins, toxin binding sites were detected in ligand blot analysis which may help to understand the reason for the observed inconsistencies between the two analyses (Figure S8). Besides this, the actual scenario becomes clearer in MD simulation where, N510A mutation has led to a great change in binding energy and compels the ligand to fly away from the binding pocket. Therefore, it can be suggest that N510 residue has an important role for GalNAc binding but its affect can be opposed by probable existence of GalNAc independent binding mechanism playing role in conferring toxicity. Therefore N510 may not be the solely important residue. These kinds of inconsistencies have been experienced by previous authors, where they justified that binding to APN receptor is not directly related to toxicity [68]. Similarly, Jenkins et al. [59] compared the toxicity and APN binding affinity of W545A mutant and observed that complete loss of APN binding caused by domain III mutation W545A leads to 50- fold decreased bio activity. Whereas, the same W545A mutation, previously characterized by Pardo-Lo´pez et al [69] showed almost similar level of biological activity against *M. sexta* with adequate receptor binding capacity. Therefore, it can be speculated that although the interaction with both the GPI-anchored receptors is mediated through the GalNAc moiety, but the insect sources being different they interacted differently. 

Apart from single mutants, the triple mutant showed drastic difference from the WT protein in terms of binding as several necessary residues around the GalNAc pocket were removed. These changes led to a 32- fold decrease in binding and an 8- fold reduction in insecticidal activity. Our findings suggests that the Q509-N510-R511 residues play a significant role in receptor binding which corroborate the findings described in previous study that showed a complete loss of binding of the triple mutant towards MsAPN-1, with 2-3 fold reduced toxicity relative to WT Cry1Ac [68,70]. Similarly, tetra mutant displays an almost 10- fold lower affinity than that of triple mutant to the HaALP receptor, further validates the crucial role of the Tyr residue in interaction in the unique GalNAc binding pocket in domain III presence of which is critical for the GalNAc-mediated mode of receptor binding.

From the biochemical data it can be interpreted that, ALP recognition is determined by domain III binding through a GalNAc moiety on the receptor. Although, our study has been focused on Cry1Ac interaction with ALP receptor at its monomeric form, but during insect bio-assay we cannot rule out the possibility of oligomer formation after the primary interaction, as the actual mode of action of Cry1Ac toxin involves sequential interaction with several insect midgut proteins facilitating the formation of a pre-pore oligomer structure and subsequent membrane insertion resulting in insect death by osmotic shock. The first binding interaction of the Cry1A monomeric toxin occurs with low affinity binding sites that concentrate more toxin molecules on the lipid raft where it binds with high affinity binding sites in its oligomeric form. But in another model it has been proposed that insect cell death is triggered by the binding of monomeric Cry1Ab toxin to cadherin receptor resulting in increased cAMP cellular levels by activation of cAMP dependant protein kinase-A resulting in cell death related to oncosis [71]. In 2006, Pardo-lopez et al. [69] showed that after oligomer formation large conformational changes occur and affinity towards APN increases almost 100 fold. Since, we have introduced mutation only at the GalNAc binding region it may so happen that after oligomer formation, mutant oligomer with defect in GalNAc binding might get affected in their further interaction with HaALP receptor. Previously, Iván Arenas, et al. [31] showed that the monomeric structure of the L511A mutant of Cry1Ab, located in domain III, was severely affected in ALP binding but with the oligomeric structure it showed a marginal effect in its interaction. Similarly, the monomeric structures of domain II loop 2 mutations had no effect on their binding to APN or ALP, whereas oligomer binding to both GPI-anchored molecules was greatly affected [31]. Therefore, it remains elusive whether the present domain III mutants follow the similar course of action through oligomer formation. Further experimentation is required for understanding this complex binding mechanism that will shed light on this complex mode of action of Cry1Ac toxin. 

## Conclusion

The present mutagenesis approach was applied on well established insecticidal protein, Cry1Ac that specified the key amino acids for the GalNAc mediated toxin-receptor interaction. Additionally, molecular modeling and simulation studies explained how these residues play important role in interaction in real time and also the detail dynamics of the binding was investigated. Significant correlation was observed between the structural studies and experimental kinetic values for different mutants in terms of their receptor binding as well as insecticidal potentiality. Such comparative study opened up a new insight of receptor binding characteristics of Cry1Ac in detail at molecular level that may help in the successful development of potent next generation toxins with altered insect specificities. 

## Supporting Information

Figure S1 **Fluorescence measurements of Cry1Ac WT and mutant toxins showing overall similar spectra for all the proteins.**
(TIF)

Figure S2 **Determination of K_d_ value from fluorescence quenching study.** The ΔF/C against ΔF was plotted and the slope (K_a_) was used to calculate the dissociation constant (K_d_) for binding of Cry1Ac to GlcNAc.
(TIF)

Figure S3 **(**A**)Time series of RMSD values were obtained for backbone atoms of WT and mutant Cry1Ac proteins.** (B) Residue wise RMSF (Å) was calculated for the WT and mutant proteins during simulation.
(TIF)

Figure S4 **Polar contacts between Cry1Ac and GalNAc molecule.** Before simulation GalNAc lies within the binding pocket and form polar contacts with the Cry1Ac molecule to strengthen the binding. Contacts around the GalNAc binding site are shown by H-bonds indicated with blue dashed lines and the residues are labeled according to their polypeptide chain and number.
(TIF)

Figure S5 **Orientation of GalNAc in tetra mutant.** Due to destabilized interaction after mutation, primary recognition of the GalNAc molecule in the WT (A) that initially forms the interacting core of this complex interaction got affected. As a result GalNAc molecule flies away from the pocket (B) that shows amino acid residues Q509-N510-R511.Y513 have a large impact in the holding of GalNAc molecule into the binding pocket.
(TIF)

Figure S6 **Determination of K_d_ value of W545A mutant for GalNAc.** The ΔF/C against ΔF was plotted and the slope (K_a_) was used to calculate the dissociation constant (K_d_) for binding of GalNAc to W545A mutant.
(TIF)

Figure S7 **Determination of K_d_ value of N510A mutant for GalNAc.** The ΔF/C against ΔF was plotted and the slope (K_a_) was used to calculate the dissociation constant (K_d_) for binding of GalNAc to N510A mutant.
(TIF)

Figure S8 **Ligand blot analysis of *H. armigera* BBMV proteins with Cry1Ac WT and N510A mutant.** Molecular weight marker (M) is indicated in left. Lane 2 and lane 3 reveals toxin binding proteins after incubation with WT and N510A Cry1Ac toxin respectively.
(TIF)

Movie S1 **Atomistic details of GalNAc binding to Cry1Ac WT protein obtained from MD simulation.** t=10 ns.
(AVI)

Movie S2 **MD simulation to characterize GalNAc binding to tetra mutant. t=10 ns.**
(AVI)

Movie S3 **Effect of single mutant N510A on GalNAc binding. t=10 ns.**
(AVI)

Movie S4 **Effect of single mutant Y513A on GalNAc binding. t= 10 ns.**
(AVI)

Table S1 **Analysis of docking results.** Various energy values of Cry1Ac- GalNAc docked complexes obtained by multiple docking runs.
(DOC)

Table S2 **Interaction energy of ligand with specific residues averaged over different window.**
(DOC)
