# Supplementary figures and images for: New Insight to Structure-Function Relationship of GalNAc Mediated Primary Interaction between Insecticidal Cry1Ac Toxin and HaALP Receptor of Helicoverpa armigera
Source: PLoS One. 2013 Oct 24;8(10):e78249. doi: 10.1371/journal.pone.0078249 (PMC3813429; doi:10.1371/journal.pone.0078249)

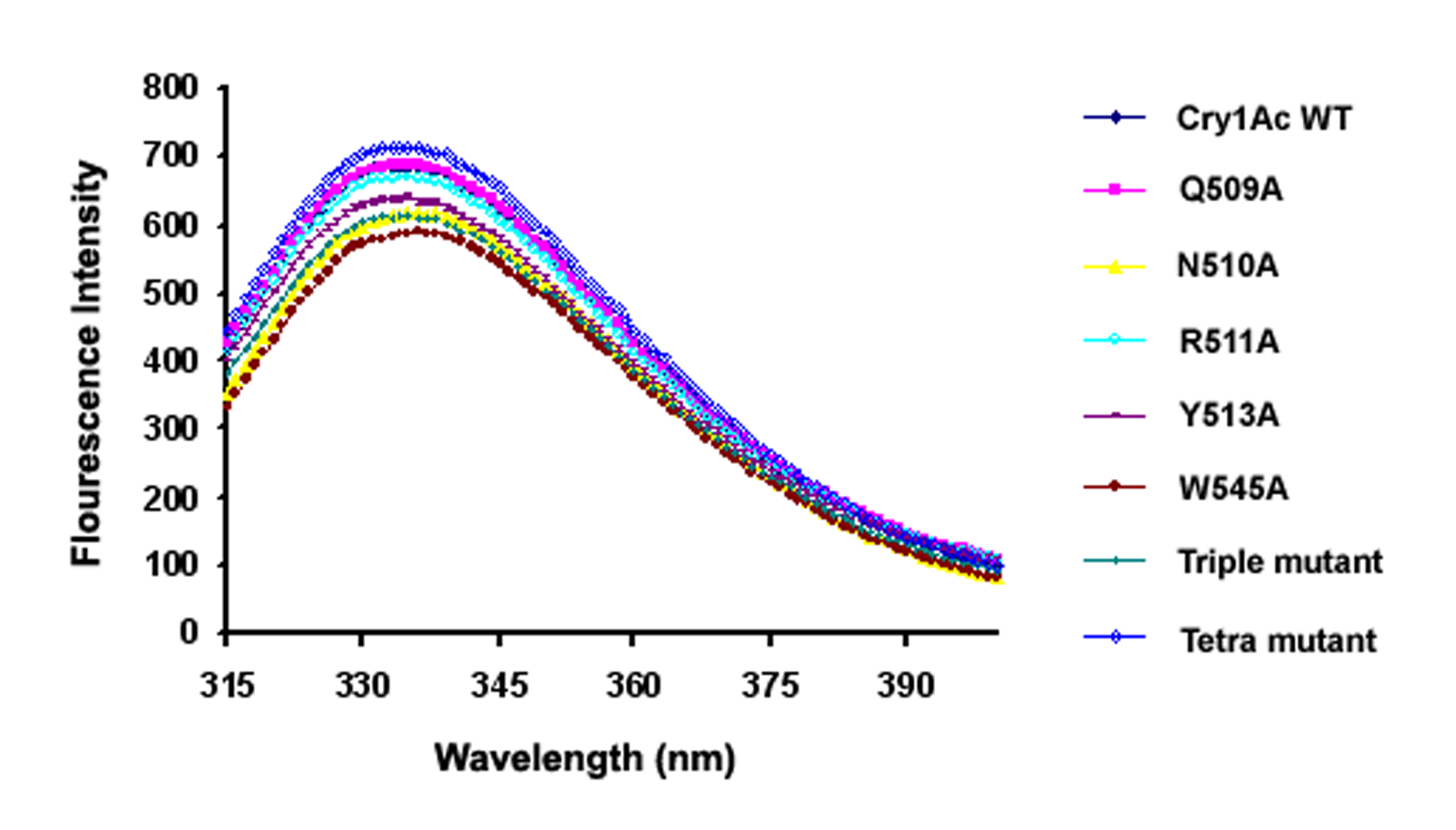

Supplement: Figure S1 — Fluorescence measurements of Cry1Ac WT and mutant toxins showing overall similar spectra for all the proteins. (TIF) [file pone.0078249.s001.tif]

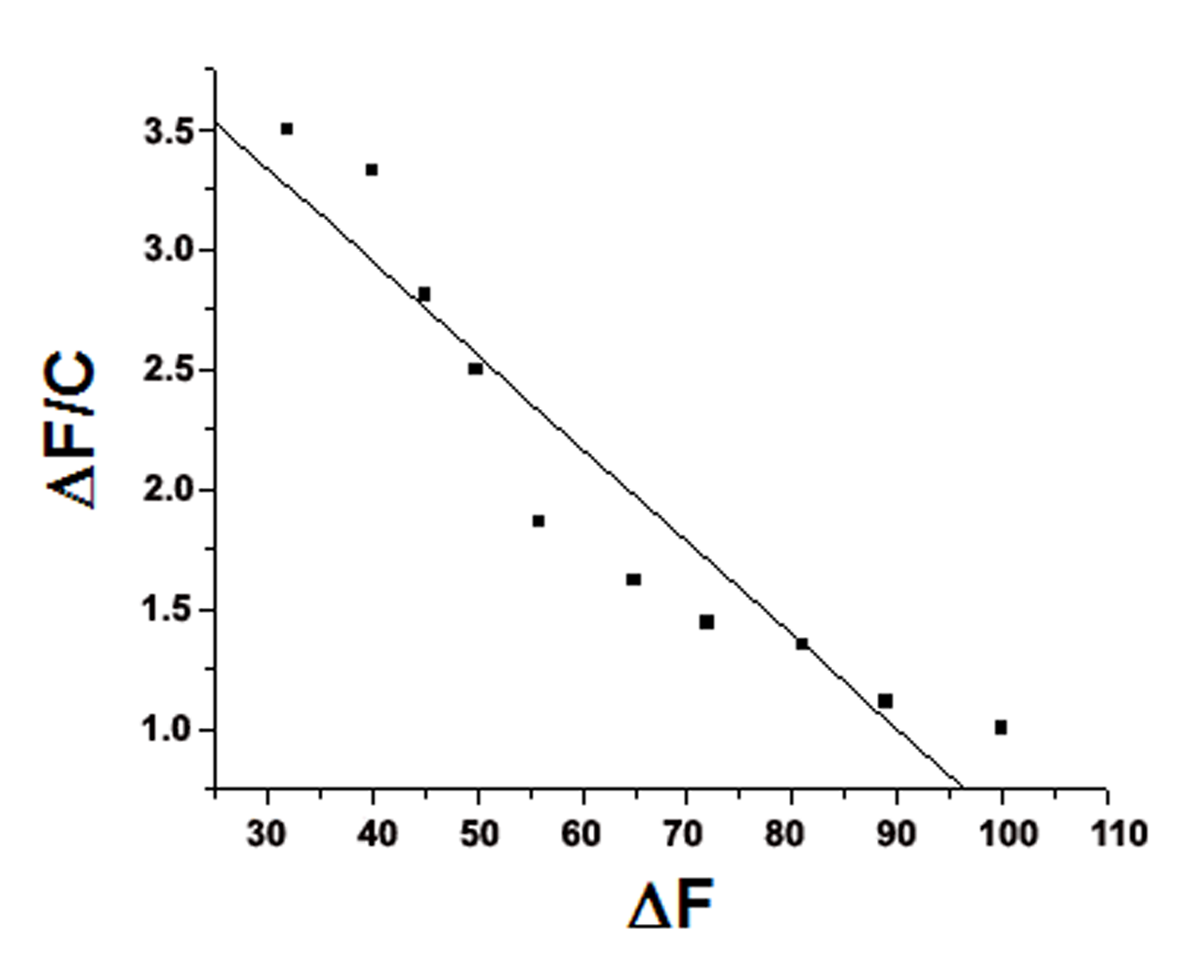

Supplement: Figure S2 — Determination of Kd value from fluorescence quenching study. The ΔF/C against ΔF was plotted and the slope (Ka) was used to calculate the dissociation constant (Kd) for binding of Cry1Ac to GlcNAc. (TIF) [file pone.0078249.s002.tif]

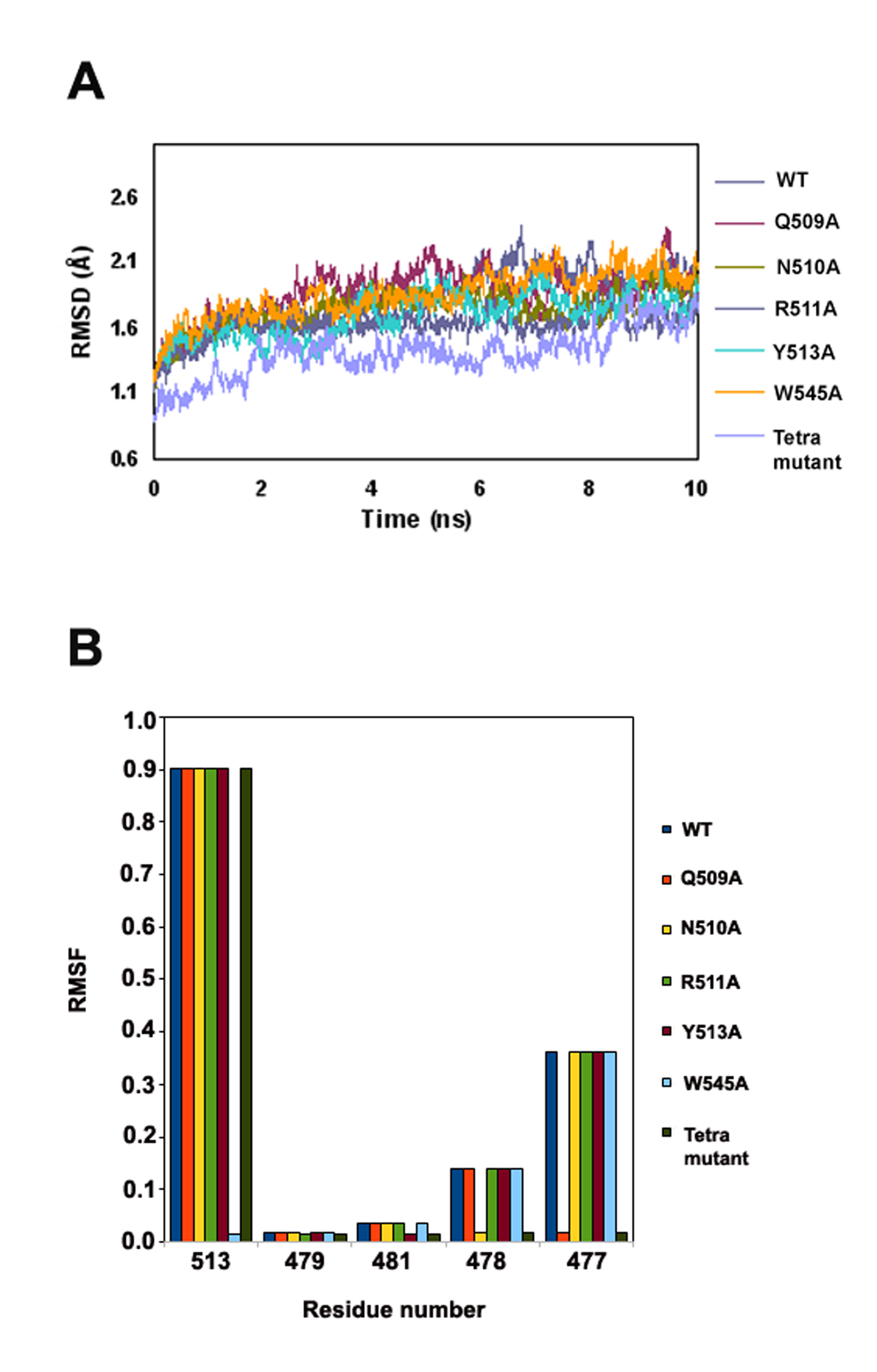

Supplement: Figure S3 — (A)Time series of RMSD values were obtained for backbone atoms of WT and mutant Cry1Ac proteins. (B) Residue wise RMSF (Å) was calculated for the WT and mutant proteins during simulation. (TIF) [file pone.0078249.s003.tif]

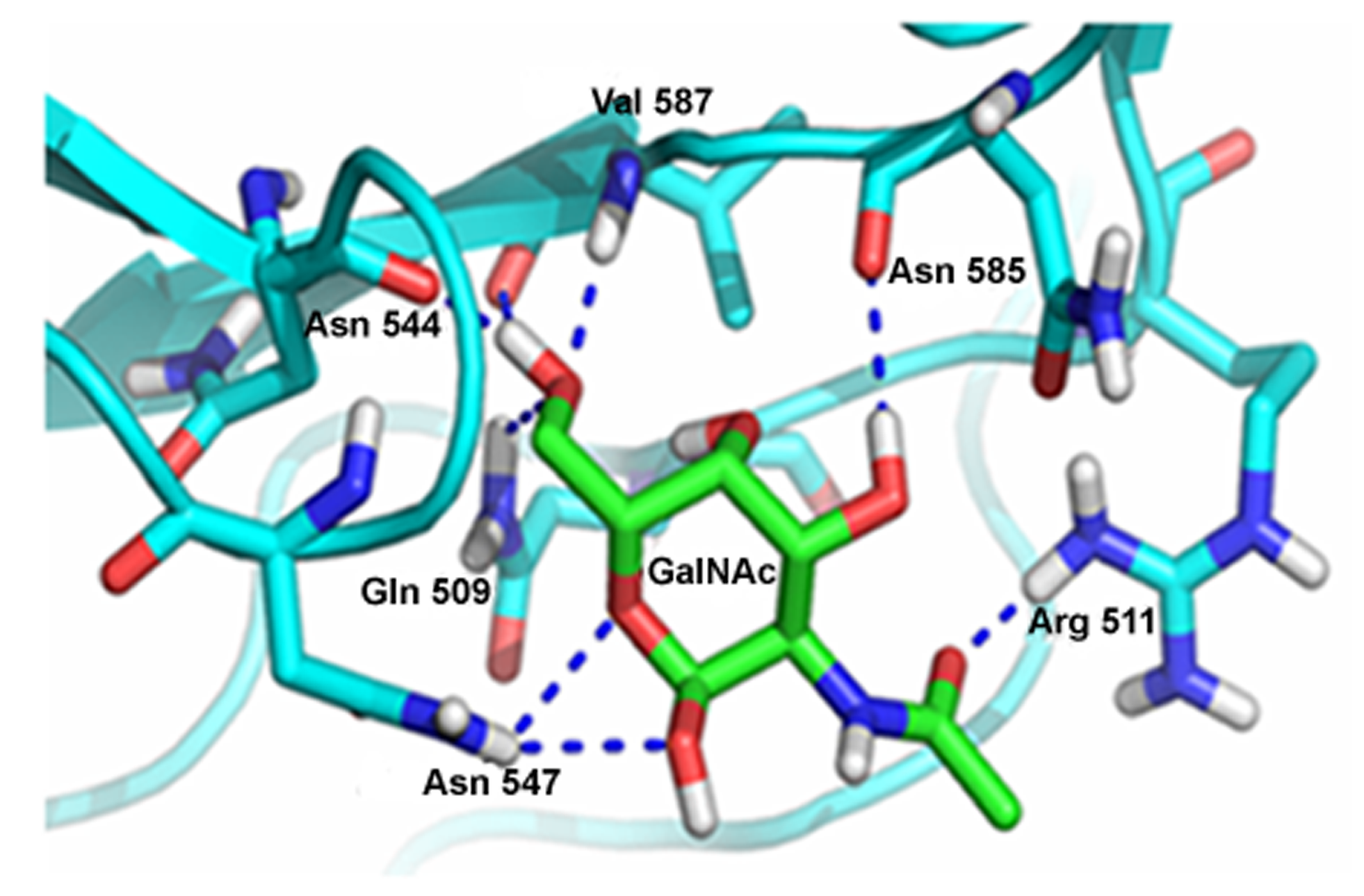

Supplement: Figure S4 — Polar contacts between Cry1Ac and GalNAc molecule. Before simulation GalNAc lies within the binding pocket and form polar contacts with the Cry1Ac molecule to strengthen the binding. Contacts around the GalNAc binding site are shown by H-bonds indicated with blue dashed lines and the residues are labeled according to their polypeptide chain and number. (TIF) [file pone.0078249.s004.tif]

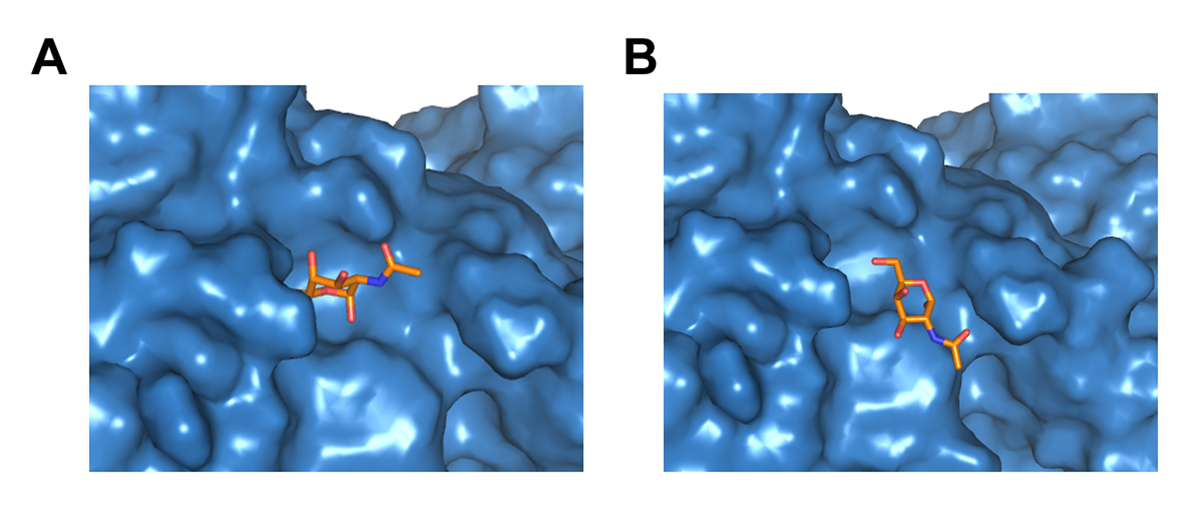

Supplement: Figure S5 — Orientation of GalNAc in tetra mutant. Due to destabilized interaction after mutation, primary recognition of the GalNAc molecule in the WT (A) that initially forms the interacting core of this complex interaction got affected. As a result GalNAc molecule flies away from the pocket (B) that shows amino acid residues Q509-N510-R511.Y513 have a large impact in the holding of GalNAc molecule into the binding pocket. (TIF) [file pone.0078249.s005.tif]

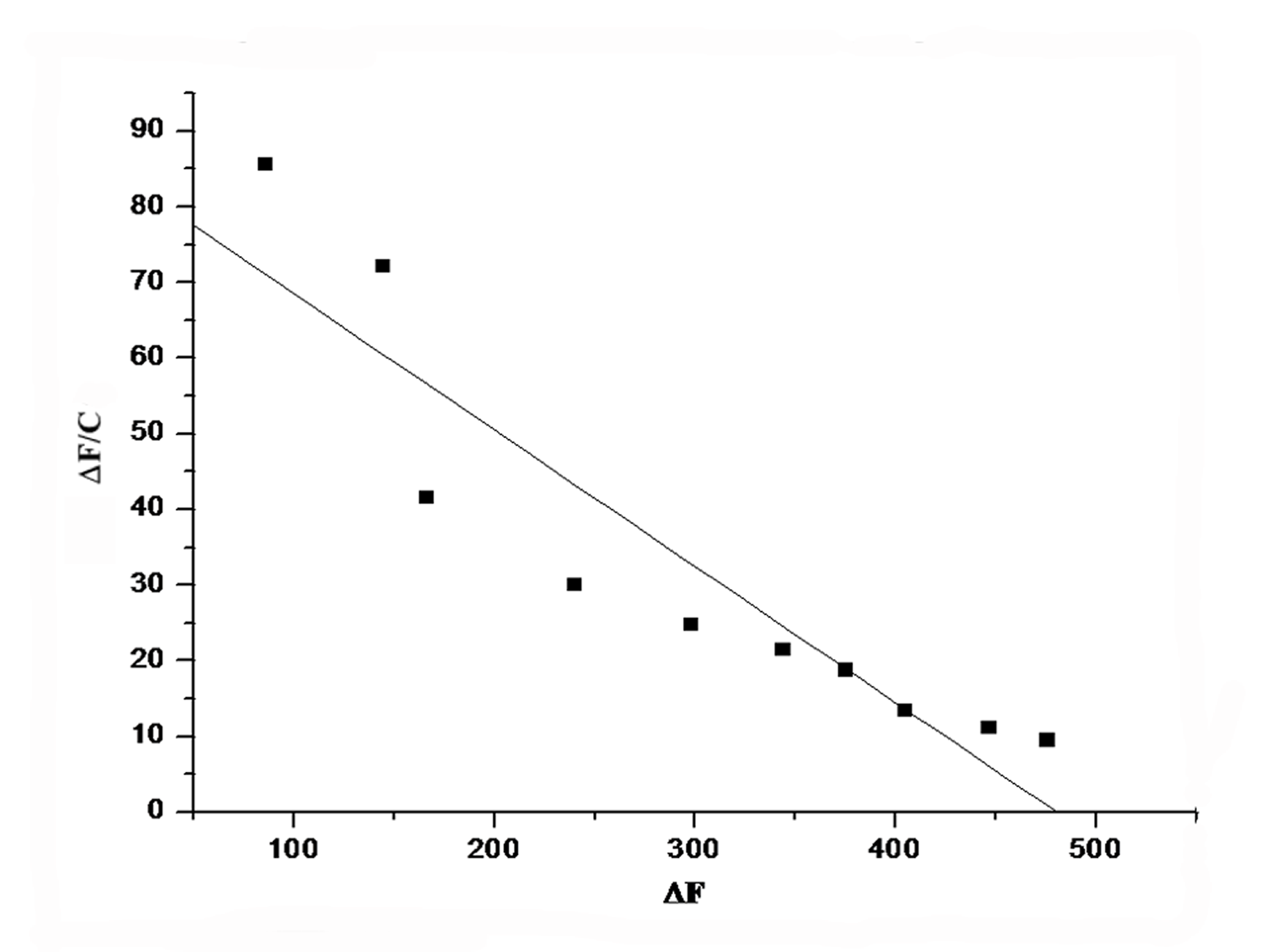

Supplement: Figure S6 — Determination of Kd value of W545A mutant for GalNAc. The ΔF/C against ΔF was plotted and the slope (Ka) was used to calculate the dissociation constant (Kd) for binding of GalNAc to W545A mutant. (TIF) [file pone.0078249.s006.tif]

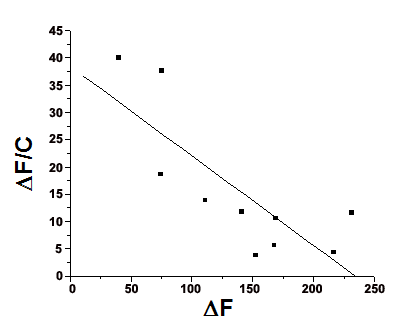

Supplement: Figure S7 — Determination of Kd value of N510A mutant for GalNAc. The ΔF/C against ΔF was plotted and the slope (Ka) was used to calculate the dissociation constant (Kd) for binding of GalNAc to N510A mutant. (TIF) [file pone.0078249.s007.tif]

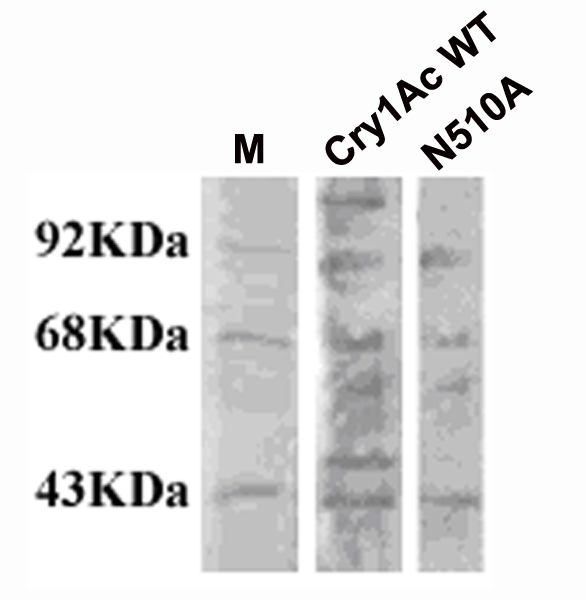

Supplement: Figure S8 — Ligand blot analysis of H. armigera BBMV proteins with Cry1Ac WT and N510A mutant. Molecular weight marker (M) is indicated in left. Lane 2 and lane 3 reveals toxin binding proteins after incubation with WT and N510A Cry1Ac toxin respectively. (TIF) [file pone.0078249.s008.tif]
