# Supplementary material for: New Insight to Structure-Function Relationship of GalNAc Mediated Primary Interaction between Insecticidal Cry1Ac Toxin and HaALP Receptor of Helicoverpa armigera
Source: PLoS One. 2013 Oct 24;8(10):e78249. doi: 10.1371/journal.pone.0078249 (PMC3813429; doi:10.1371/journal.pone.0078249)
Supplement: Table S1 — Analysis of docking results. Various energy values of Cry1Ac- GalNAc docked complexes obtained by multiple docking runs. (DOC) [file pone.0078249.s013.doc]

**Supplemental Table S1**

| Autodock Run | Binding Energy (kcal/mol) | InhibitionConstant | Final Inter-molecular Energy (kcal/mol) | VDw+Hbond+DesolvationEnergy (kcal/mol) | Electrostatc Energy (kcal/mol) | Final Total Interaction Energy (kcal/mol) |
| --- | --- | --- | --- | --- | --- | --- |
| 1. | -4.20 | 834.72µM | -4.36 | -4.24 | -0.12 | -2.22 |
| 2. | -3.81 | 1.61mM | -4.30 | -4.13 | -0.16 | -1.89 |
| 3. | -3.63 | 2.19mM | -3.58 | -3.48 | -0.09 | -2.42 |
| 4. | -4.20 | 836.46µM | -4.25 | -4.19 | -0.06 | -2.32 |
| 5. | -4.31 | 695.09µM | -4.84 | -4.66 | -0.18 | -1.84 |
| 6. | -3.80 | 1.65mM | -4.30 | -4.20 | -0.11 | -1.86 |
| 7. | -4.16 | 895.57µM | -4.57 | -4.49 | -0.08 | -1.95 |
| 8. | -3.91 | 1.36mM | -4.10 | -3.97 | -0.13 | -2.18 |
| 9. | -4.22 | 808.57µM | -4.50 | -4.44 | -0.06 | -2.09 |
| 10. | -4.21 | 817.60µM | -4.98 | -4.78 | -0.21 | -1.60 |
