# Supplementary material for: New Insight to Structure-Function Relationship of GalNAc Mediated Primary Interaction between Insecticidal Cry1Ac Toxin and HaALP Receptor of Helicoverpa armigera
Source: PLoS One. 2013 Oct 24;8(10):e78249. doi: 10.1371/journal.pone.0078249 (PMC3813429; doi:10.1371/journal.pone.0078249)
Supplement: Table S2 — Interaction energy of ligand with specific residues averaged over different window. (DOC) [file pone.0078249.s014.doc]

**Supplementary Table S2**

| Residue | 0-1^st^ ns | 1^st^-2^nd^ ns | 2^nd^-3^rd^ ns | 3^rd^-4^th^ ns | 4^th-5th^  ns | 5^th^-6^th^ ns | 6^th^-7^th^ ns | 7^th^-8^th^ ns | 8^th^-9^th^ ns | 9^th^10^th^ns |
| --- | --- | --- | --- | --- | --- | --- | --- | --- | --- | --- |
| Gln509 | -5.80 | -4.45 | -4.32 | -4.55 | -3.09 | -3.01 | -1.67 | -0.32 | -2.44 | -2.92 |
| Asn510 | -2.34 | -2.07 | -2.85 | -3.24 | -3.33 | -2.72 | -1.68 | -0.20 | -0.54 | -0.74 |
| Arg511 | -15.31 | -12.45 | -12.08 | -14.46 | -15.32 | -5.64 | -2.37 | -0.58 | -2.59 | -6.52 |
| Tyr513 | -0.11 | -0.05 | -0.06 | +0.01 | +0.04 | -0.03 | -0.04 | -0.00 | -0.02 | -0.01 |
| Trp545 | -1.60 | -1.71 | -0.92 | -0.93 | -0.84 | -0.22 | -0.11 | -0.11 | -0.31 | -0.32 |
